# Defining Clonal Color in Fluorescent Multi-Clonal Tracking

**DOI:** 10.1038/srep24303

**Published:** 2016-04-13

**Authors:** Juwell W. Wu, Raphaël Turcotte, Clemens Alt, Judith M. Runnels, Hensin Tsao, Charles P. Lin

**Affiliations:** 1 Wellman Center for Photomedicine, Massachusetts General Hospital, Harvard Medical School, Boston, MA 02114, USA; 2 Center for Systems Biology, Massachusetts General Hospital, Harvard Medical School, Boston, MA 02114, USA; 3 Department of Dermatology, Harvard Medical School, Boston, MA 02114, USA; 4 Wellman Center for Photomedicine, Harvard Medical School, Boston, MA 02114, USA; 5 Massachusetts General Hospital Melanoma and Pigmented Lesion Center, Boston, MA 02114, USA; 6 Harvard Stem Cell Institute, Cambridge, Massachusetts 02138, USA

## Abstract

Clonal heterogeneity and selection underpin many biological processes including development and tumor progression. Combinatorial fluorescent protein expression in germline cells has proven its utility for tracking the formation and regeneration of different organ systems. Such cell populations encoded by combinatorial fluorescent proteins are also attractive tools for understanding clonal expansion and clonal competition in cancer. However, the assignment of clonal identity requires an analytical framework in which clonal markings can be parameterized and validated. Here we present a systematic and quantitative method for RGB analysis of fluorescent melanoma cancer clones. We then demonstrate refined clonal trackability of melanoma cells using this scheme.

Long-term and non-destructive tracking of live cells has been made possible with fluorescence imaging and endogenous cell labeling with fluorescent proteins (FP). Development of engineered animal models expressing combinatorial copies of stochastically chosen fluorescent protein reporters, most notably the “Brainbow” mouse^1^,^2^ and its successors, has permitted fluorescent cell and clonal tracking *in vivo* and *ex vivo* with their ability to distinctively color-code multiple cells and clones. Real-time observation of clonal interactions at single cell resolution informs on clonal dominance, extinction and changes in spatial resolution and has offered invaluable insight to developmental, regenerative and cancer biology^3^,^4^,^5^,^6^,^7^,^8^,^9^,^10^,^11^,^12^,^13^,^14^,^15^,^16^,^17^,^18^,^19^,^20^,^21^,^22^,^23^,^24^,^25^,^26^,^27^,^28^,^29^,^30^,^31^.

Rapid expansion in FPs’ spectral repertoire, improvements in their photochemical properties and tolerated expression levels have created a rich palette for simultaneous tracking of multiple cells and clones. In response to the increasingly crowded color space, cell color descriptors in literature have evolved from broad, colloquial terms dependent on human vision (“purple”) to RGB quantifiers, hue and saturation values^1^,^10^,^16^,^23^,^27^,^32^,^33^,^34^,^35^. Nonetheless, clonal identity, as defined by the collective RGB properties of cells in a clone, has yet to be described. Fluorescent clonal tracking operates on clonal RGB descriptor rather than individual cell RGB descriptor. If a cell is represented by a point in the RGB space, a clone is the collection of points that occupy a finite volume in the RGB space. Knowledge of clonal RGB properties is a requisite for matching individual cells to their clonal origin during clonal tracking studies. Sophisticated clustering algorithms, which have seen use for this task of clonal assignment^27^, must make implicit assumptions on how cell colors are distributed in participant clones. Assignment accuracy of these algorithms is hence limited by the accuracy of these assumptions.

Our goal is to devise a strategy for fluorescent clonal tracking such that each individual cell can be rigorously tracked back to its clonal origin, independent of human vision subjectivity or statistical models, and spatial and morphological attributes of clonal cells. Towards this goal, we will first perform a large scale study of “Rainbow” clones, each with a combinatorial expression of three fluorescent proteins, and define metrics for the clonal RGB properties that most influence the setup and interpretation of fluorescent clonal tracking experiments. We will then describe the criteria for selecting clones suitable for fluorescent clonal tracking using these metrics and construct the quantitative framework for clonal assignment. We will finally demonstrate the efficacy of our method by establishing a human melanoma cell line population with verifiable clonal trackability and report its clonal composition for fifteen weeks. Based on our findings, we will introduce a new strategy that allows robust clonal tracking in live cells, relying solely on fluorophore expression as the clonal marker.

## Results

### Defining the color space

Color descriptions, whether of cells or clones, are only meaningful when referenced to a well-defined, consistent color space. The color space must also accommodate all cell colors that may be presented. For ease of communication, “colors” in this manuscript will from here on refer to RGB combinations.

**Figure 1:**
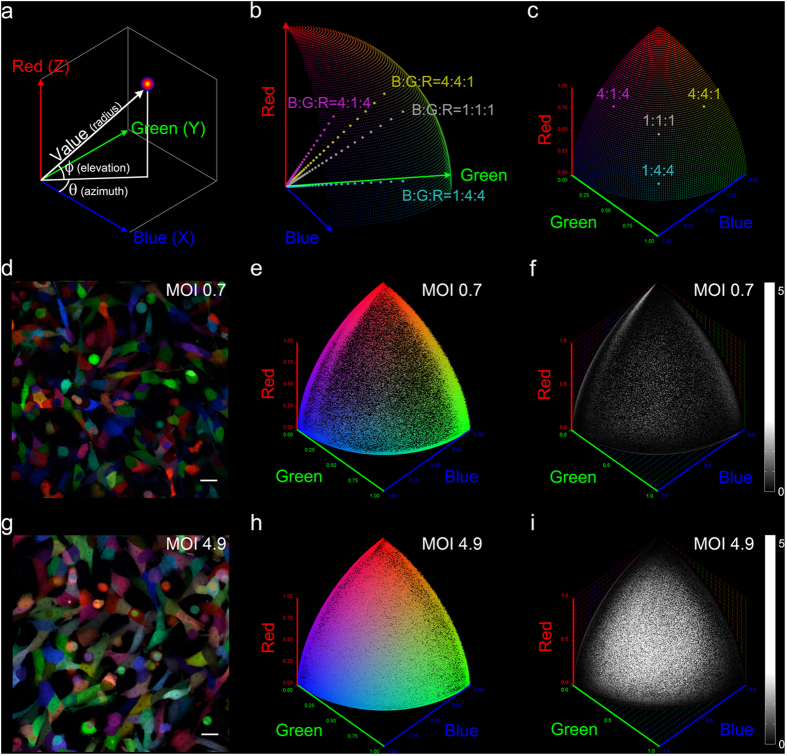
Cell color and the chromaticity grid. (**a**) RGB cell colors in spherical coordinates. Azimuth Θ and elevation Φ describe chromaticity. Radius describes brightness, or value. (**b**) Cells of equal chromaticity, regardless of value, project to the same point on the first octant surface of a sphere, which we defined as the chromaticity grid. (**c**) 1FP+ and 2FP+ expressions are positioned at the corners and edges of the chromaticity grid, respectively. 3FP+ expressions are positioned on the grid. (**d**) Representative confocal image of MelaChroma, MOI = 0.7, 32 days after lentiviral transduction. (**e**) Spherical scatter plot of (**d**). Each data point, at chromaticity coordinate (Θ, Φ), designates the chromaticity value of a cell (2.6E5 total, analyzed by flow cytometry). (**f**) Spherical histogram of (**d**). Each grid element (Θ = 0.2° × Φ = 0.2°) displays the adjusted cell count “binned” to the element (Supplementary Fig. S1). (**g**) Representative confocal image of MelaChroma, MOI = 4.9, 32 days after lentiviral transduction. (**h**) Spherical scatter plot of (**g**). 2.6E5 cells were analyzed. (**i**) Spherical histogram of (**g**). Compared to (**f**), this higher MOI population was enriched in 3FP-expressing cells. Scale bar: 25 μm.

First, we developed a system for quantifying cell colors that separates the ratio versus the amplitude of fluorescence intensity signal in Red, Green and Blue. Contributors of fluorescence include both the fluorescent proteins and autofluorescence. We converted the 3D Cartesian RGB fluorescent signal intensities into spherical coordinates and defined chromaticity, in azimuth Θ and elevation Φ, as the value-normalized color (Fig. 1a). Cells with the same R:G:B intensity signal ratio in the color space project to the same chromaticity coordinate (Θ_0_, Φ_0_) on our chromaticity grid, which is easily visualized with the surface of the first octant of a sphere (Fig. 1b,c).

Maximum color diversity is achieved when the full range of intracellular concentrations of FPs well tolerated by cells are present in a cell population. To determine the widest, viable range of FP concentrations, we performed the RGB cell marking scheme using lentiviral gene ontology (LeGO) vectors developed by Weber *et al*.^36^,^37^,^38^ on five A375 human amelanotic melanoma cell line populations. We transduced each population with three LeGO lentiviral particle constructs carrying the genetic code of Cerulean^39^ (CFP variant, assigned to Blue), Venus^40^ (YFP variant, Green) and tdTomato^41^ (RFP variant, Red) at equal dosages of multiplicity of infection (MOI) 0.7, 1.4, 2.8, 4.9, and 7.0 respectively. We named these cell populations MelaChroma for their visually colorful palette (Fig. 1d,g).

We introduced spherical scatter plots (Fig. 1e,h) and histograms (Fig. 1f,i, Supplementary Fig. S1) to display chromaticity information for both monoclonal populations and multi-clonal populations such as the MelaChromas. Cells fluorescing in one of the three primary chromaticities (red, green, blue), i.e., those expressing a single FP (1FP+), congregate at the corners of these plots. Cells expressing any combination of two FPs (2FP+) fluoresce in two of the three primary chromaticities (red+green, green+blue, blue+red) and congregate along the edges. Cells expressing all three FPs (3FP+) spread over the plots’ surface. Spherical scatter plots and histograms of the MelaChomas revealed a balanced presence of blue, green and red FPs and improved chromatic diversity at higher MOIs, the latter evidenced by the filling of the surface of the chromaticity grid that signified increased percentage of 3FP+ cells (Fig. 1d–i, Supplementary Fig. S2). Such enhancement in chromatic diversity, predicted by Poisson statistics^38^,^42^, nonetheless diminished by MOI 7.0, which also showed no improvement in fluorescent signal intensities compared to MOI 4.9 (data not shown). Higher lentiviral dosages were not pursued.

Next, we established and fixed the acquisition parameters (such as laser excitation power and detector gain) for all systems to be used for clonal tracking with the same cell populations. We imaged and performed flow cytometry on the five MelaChroma populations, which would supply the clonal founder cells. Following the previous recommendations for “Brainbow” imaging^14^,^31^, the settings of our fluorescence confocal microscope and flow cytometer were adjusted such that each detection channel, in each imaging system, spanned the available intensity scale with minimal saturation (0 to 65535 (16-bit) for fluorescence microscope, 0 to 1E5 for flow cytometer). Cell colors in confocal images therefore matched the colors reported by flow cytometry (Fig. 1d,e,g,h, Supplementary Fig. S3). We also kept these imaging system settings constant throughout the study to obtain consistent color reporting over time.

### Generating clonal populations

**Figure 2:**
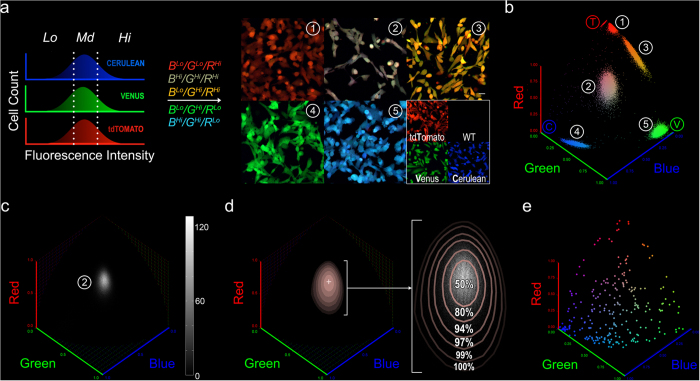
Clonal founder cell selection, clonal chromatic mode and chromatic spread. (**a**) Combinatorial gating scheme for single clonal founder cell sorting. RGB channels were gated by intensity and different combinations of these R/G/B channel gates were used to select single cells from MelaChromas for clonal expansion. MelaChromas of various MOIs (0.7, 2.8, 4.9) were used the source of founder cells. As a result, a wide variety of FP expression levels and Cerulean(B):Venus(G):tdTomato(R) ratios were represented in the selected cells, as shown in the confocal images of expanded clones. For visual reference, confocal images of 1FP-expressing clones and un-transduced A375 cells (A375-WT) at 50% magnification are also shown. Scale bar: 25 μm. (**b**) Spherical scatter plots of the clones in (**a**). 1E4 cells were analyzed by flow cytometry per clone. (**c**) Spherical histogram of clone #2 in (**a,b**). (**d**) Chromatic mode (crosshair) and chromatic spreads (pink) of clone #2. Chromatic spreads, as projections of isosurfaces drawn at 50%, 25%, 10%, 5%, 2%, and 1% of the highest cell count at the chromatic mode (Supplementary Fig. S4), co-localized with the high cell count region in the clonal spherical histogram (same as (**c**), shown magnified in white). Values indicate the actual percentage of clonal cells with chromaticity values enclosed within each contour. (**e**), Spherical scatter plot showing the chromatic mode coordinates of the 256 expanded MelaChroma clones.

We devised a strategy to isolate chromatically diverse founder cells for clonal expansion. Our goal was to build a library of diversely color-coded clones with representative clonal color properties, which would also serve as potential participant clones in a multi-clone tracking study. *In vitro* cultures of individual clones and flow cytometry allow repeatable clonal color data collection at high cell counts per clone. We used MelaChroma cells of MOIs 0.7, 2.8 and 4.9 as our source of founder cells. These MelaChromas had different distributions of intracellular FP concentrations, corresponding to different RGB intensities. To avoid oversampling of high frequency chromaticities in each MelaChroma, we created a combinatorial FACS gating scheme for founder cell sorting that further ensured selection of cells with diverse RGB ratios (Fig. 2a,b).

### Defining metrics for characterizing clonal colors

In an ideal clonally trackable population, each cell can be unambiguously assigned to one of the pre-determined list of participating clones. This requires the following color criteria to be met^16^,^27^,^33^: 1) the color of each participant clone is distinguishable from other participant clones’; 2) minimal contribution of autofluorescence to the color code, in this case by the fluorescent proteins; and 3) the color of each participant clone is stable for the duration of the clonal tracking experiment.

Based on these criteria, we defined four metrics for clonal color: chromatic mode, chromatic spread, relative clonal brightness and chromatic stability. Chromatic mode and spread measure the chromaticity center of a clonal population and the span of its chromaticity distribution. Relative clonal brightness measures the relative contribution of fluorescent proteins against autofluorescence towards total clonal fluorescence intensity. Chromatic stability measures clonal chromatic shifts over time. Robust relative clonal brightness and chromatic stability qualifies individual clones as candidates for long term clonal tracking. Chromatic mode and chromatic spread then limits the number of qualified clones that are distinguishable and can participate in a clonal tracking study (Supplementary Fig. S4).

We then provided analytical definitions for our metrics, in the context of our color space and chromaticity grid. We designated a clone’s chromatic mode as the chromatic coordinate with the highest clonal cell count *n*_*max*_. We quantified the chromatic spread as the area confined within the chromaticity grid projection of fractional *n*_*max*_ isosurface (Fig. 2c,d, Supplementary Fig. S5).

Our relative clonal brightness metric reports the percentage of clonal cells with FP-to-autofluorescence contribution to total cellular fluorescence surpassing a benchmark value b^*^. We analyzed the autofluorescence (AF) of un-transduced A375 cells and showed that it behaved like clonal colors with characteristic chromatic mode, chromatic spread and value (Supplementary Fig. S5). Cells with diminishing FP-to-AF contribution chromatically skew towards the chromatic mode of autofluorescence. Consequently, clones with subpopulations of these “dim” cells could suffer from very large chromatic spreads. As AF was used as reference for comparing FP fluorescence contribution, our measurement of relative clonal brightness began with the definition of one autofluorescence unit, or 1 xAF (Multiple of Autofluorescence), with the 2% isosurface chromatic spread of the un-transduced A375 population. We then defined the relative brightness of individual cells, regardless of clonal origin, as *b* multiples of 1 xAF; autofluorescence contribution to the cell’s total fluorescence is expected to be <*1/b* (Supplementary Fig. S5). Relative clonal brightness was subsequently defined as the fraction of clonal cells that surpass a sufficiently high b^*^ value at which autofluorescence no longer influence clonal chromatic spreads. Ideal relative clonal brightness is one.

We measured chromatic stability by quantifying the change in clones’ chromatic spread over time (Supplementary Fig. S6). Conceptually, our calculation for chromatic stability reports the additional area covered by a clone’s chromatic spread per time point measurement with respect to the clone’s smallest single-time-point chromatic spread area. The unitless outcome is hence insensitive to the size of the chromatic spread, which allows inter-clonal comparison. Ideal chromatic stability is zero, meaning there is no gain in chromatic spread areal coverage.

We then applied the four clonal color metrics to characterize our 256 expanded MelaChroma clones. We confirmed our collection of clones was chromatically representative of the full chromaticity grid by plotting the clones’ chromatic modes (Fig. 2e). However, we observed non-uniformity in the clones’ chromatic spread and chromatic stability, which followed a roughly Gaussian distribution with a small but significant number of poorly performing outliers but no chromaticity dependence (Supplementary Fig. S7). Relative brightness of cells within a clone typically followed a skewed xAF distribution that is cell cycle status dependent (Supplementary Fig. S8); clones with inferior relative clonal brightness congregated near the blue corner of the chromaticity grid (Supplementary Fig. S7).

### Selecting chromatically distinguishable clones; creating clonally trackable MelaChroma and clonal assignment

Accuracy of clonal assignment is inversely related to the amount of overlap between chromatic spreads of participant clones. Accuracy is guaranteed when there is no overlap, but at the cost of fewer clones being able to participate. We therefore opted to examine a more realistic scenario in which minor overlaps of chromaticity spread are allowed and devised a method to estimate the assignment error associated with such overlaps.

Our scheme for clonal assignment was to match the chromaticity coordinate of each cell of unknown clonal origin to the known set of chromaticity coordinates bounded by each participant clone’s chromatic spreads. We constructed the matching template, or chromatic landscape that described the chromatic mode (“peaks”) and chromatic spreads (“altitudes” in cell count) of all participant clones, using computational analysis of the clonal color data collected from the previous section. First, we finalized our candidate clonal pool by admitting only MelaChroma clones that met the criteria of non-outlying chromatic stability and 97.5 percentile for relative clonal brightness, with b* set at 20. Higher b* values improve clonal identification accuracy but admit fewer clones into the pool (Supplementary Fig. S9).

**Figure 3:**
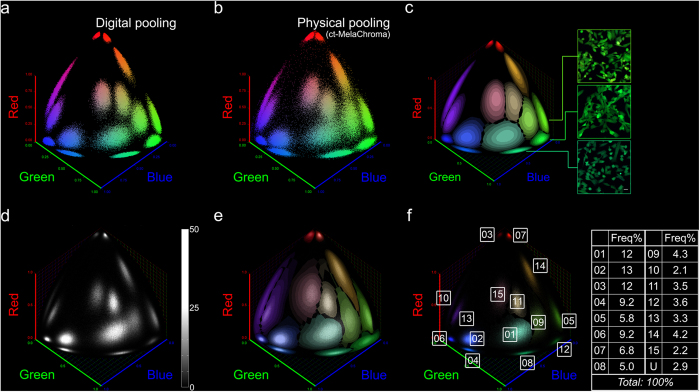
Generating clonally trackable MelaChroma and clonal assignment. (**a**) Digitally pooled color data of a 15-clone subset selected from the MelaChroma candidate clonal pool. These fifteen clones had non-overlapping chromatic modes and 50% isosurface chromatic spreads. Color data of 9E4 cells represented each clone. Spherical scatter plot of the pooled color data is shown. (**b**) Spherical scatter plot of the cell population generated by physically pooling the fifteen clones in (**a**). We named this population ct-MelaChroma for its clonal trackability. 1.35E6 cells were analyzed by flow cytometry two days after clonal pooling. (**c**) Chromatic landscape of ct-MelaChroma. Chromatic modes and chromatic spreads of the fifteen clones were positioned on this landscape by the digital pooling of clonal color data (**a**). Clonal assignment rules for chromaticity grid elements mapped to multiple clones were subsequently applied (Supplementary Fig. S10). Confocal images exemplify clones that were difficult to distinguish by eye but readily distinguishable by chromaticity plotting (scale bar: 25 μm). (**d**) Spherical histogram of ct-MelaChroma eight days post clonal pooling. ct-MelaChroma’s flow cytometry data carried no information of each cell’s clonal identity. 1.53E6 cells were analyzed by flow cytometry. (**e**) Our clonal assignment algorithm numerically matched the chromaticity coordinate of each ct-MelaChroma cell to the chromaticity coordinates bounded by each clone in the chromatic landscape. An overlay of (**d**,**c**) illustrates this concept. Correction of non-ideal chromatic stability had been performed (Supplementary Fig. S11). (**f**) ct-MelaChroma’s clonal composition is the output of the clonal assignment algorithm. Cells in the spherical histogram (**d**) were color-coded according to their assigned clone. Numerical frequency of each participant clone was listed. “U” (unassigned) designated cells with out-of-bounds chromaticity coordinate in the chromatic landscape or cells that could not be exclusively assigned to one clone (“yellow” region, Supplementary Fig. S10).

Next, we digitally pooled the color data of different subsets of clones from this candidate pool to determine a subset for actual mixing, with inter-clonal distinguishability based on their chromatic mode and chromatic spreads (Fig. 3a). Our elected subset of fifteen clones exhibited non-overlapping chromatic mode and 50% isosurface chromatic spread and we named the resulting multi-clonal population ct-MelaChroma for its clonal trackability. Chromaticity plot of the physically pooled fifteen clones of ct-MelaChroma (Fig. 3b) agreed well with its digitally pooled model.

Our chromatic landscape also served as an efficient visual and computational framework for addressing the uncertainty of assignment for (Θ, Φ) chromaticity coordinates mapped to multiple clones (Supplementary Fig. S10). We reasoned that given the % isosurface of overlapping chromatic spreads at a chromaticity coordinate (Θ_0_, Φ_0_) describe the relative occupation probability of the corresponding clones, if a cell at (Θ_0_, Φ_0_) must be assigned to one of the clones, then the relative occupation probability must also describe the probability of accurate clonal assignment. Application of these clonal assignment rules (Supplementary Fig. S10) created our finalized ct-MelaChroma’s chromatic landscape, which also confirmed chromaticity measurements as powerful discriminators of clones that were visually similar in color (Fig. 3c).

To validate the utility of the chromaticity landscape, we performed clonal assignment on each of the fifteen participant clones of ct-MelaChroma. As each cell’s clonal origin was known, we were able to confirm that >86% cells in each clone were correctly assigned. We were also able to determine the percentage of cells in each clone that were “Unassigned” based on our clonal assignment rules and that were assigned to a wrong clone. Re-arrangement of these results created the spillover matrix M_s_, specific to clonal assignment rules and participant clones’ chromatic properties. M_s_ reports the relative occupation probabilities and is used for calculating clonal assignment accuracy (Supplementary Fig. S10). M_s_ for ct-MelaChroma predicted a clone-dependent assignment error range of [0.45%, 7.11%].

### Long-term clonal tracking of clonally trackable MelaChroma

To examine the long-term efficacy of ct-MelaChroma, we observed the clonal composition of ct-MelaChroma’s for fifteen weeks. Two days after ct-MelaChroma pooling, we distributed the cells into six independent samples and determined the participant clonal frequencies in each sample over time.

We developed an algorithm for automatic clonal assignment of ct-MelaChroma cells (Fig. 3d,e). Chromaticity mismatches due to non-ideal clonal chromatic stability were corrected prior to clonal assignment (Supplementary Fig. S11). The algorithm’s output was ct-MelaChroma’s clonal composition, a list of frequencies of the fifteen participant clones (Fig. 3f). Clones at <5% frequency can be recovered by this algorithm even when the chromatic landscape is incomplete and M_s_ remains undefined (Supplementary Fig. S12).

**Figure 4:**
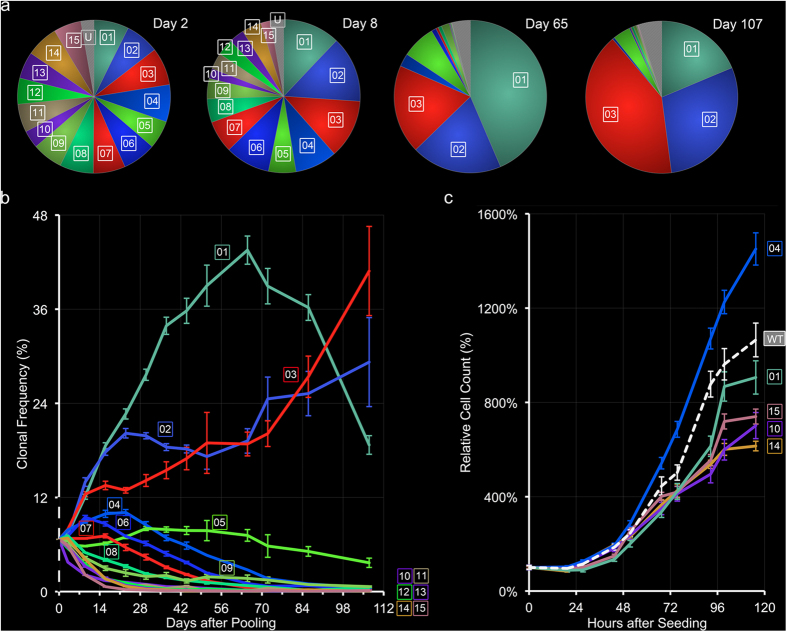
ct-MelaChroma’s clonal composition over time. (**a**) Mean frequency of participant clones of ct-MelaChroma on various days post pooling. Emergence dominance of three clones (01, 02, 03) was evident by Day 8. (**b**) Clonal composition of ct-MelaChroma over the course of fifteen weeks. The 12% clonal frequency cutoff at Week 2 separated the clones that trended towards dominance (clones 01–3) versus withdrawal (clones 04–15). Error bars denote standard deviations of the six samples analyzed by flow cytometry for the day except for Days 51 and 72, on which two samples were analyzed. 1.35–2.32E6 cells were clonally assigned, per sample, on all days. (**c**) Mono-clonal growth curves of five of ct-MelaChroma’s participant clones. WT denotes un-transduced A375 cells. Four measurements were made per time point between two consecutive sub-cultures. Error bars indicate standard errors for each measurement.

Clonal frequencies showed small standard deviations between the six ct-MelaChroma samples, particularly at early time points. Divergence of ct-MelaChroma’s participant clonal frequencies from their initial, matching values (100/15 = 6.67%) occurred early and quickly (Fig. 4a,b). Three dominant clones emerged (01, 02, 03), roughly doubling in clonal frequency in the first two weeks, while other clones (04–15) trended towards withdrawal. Notably, the most dominant clone (01) in the first half of the study exhibited frequency decline beyond Week 9. To determine if the observed pattern of clonal evolution is simply a reflection of individual clonal growth rate, we measured the growth of five participant clones in mono-clonal cultures (Fig. 4c). Interestingly, the fastest growing clone (04) was a withdrawing clone in ct-MelaChroma and its growth rate surpassed that of ct-MelaChroma’s dominant clone 01.

## Discussion

We presented a method for performing fluorescent clonal tracking by characterizing clonal colors from triple fluorescent protein expression. By using an *in vitro* cell line, we were able to identify with certainty the progenies of individual clonal founder cells and perform large-scale sampling that allowed us to define proper metrics for clonal colors. These metrics streamlined the collection of *a priori* clonal color information, cumulating to the construction of a deterministic clonal assignment scheme for multi-clonal tracking. We were thus able to generate a multi-clonal population with chromaticity distinguishable clones, validate its clonal trackability, and report its clonal composition over time with known accuracy.

We observed a remarkably consistent pattern of clonal evolution among the six duplicates of our fifteen-clone ct-MelaChroma population. Clonal frequencies over the fifteen weeks showed small sample-to-sample deviations, with the same subsets of participant clones trending towards dominance and withdrawal. While variations in participant clonal growth rates could explain the outcome, our data (Fig. 4c) suggested additional driving forces related to the complex clonal interactions in the tumor microenvironment. These driving forces may be related to interclonal Darwinian selection pressures, as recently summarized by Tabassum and Polyak^43^.

Fluorescent clonal tracking operates on clonal color. Clonal color codes are identified not by the color of a cell, but the color of the ensemble of cells belonging to a clone. Our study highlights the importance of independent verification of clonal diversity when fluorescence color is utilized as a clonal identifier: cell color diversity, when observed in a multi-clonal population, infers clonal diversity only when certain properties about clonal color are known. We described these properties in four metrics: chromatic mode, chromatic spread, relative clonal brightness and chromatic stability. These metrics quantify the criteria for building a clonally trackable population: clonal inter-distinguishability, minimal autofluorescence contribution to clonal fluorescence and stability of clonal color over time.

A database of clonal color properties based on the four metrics provides a platform for creating multiple clonally trackable populations from the same candidate pool, perform reliable *in vitro* fluorescence clonal tracking with each at known accuracy. Similar databases as our MelaChroma database can provide insights to the expected clonal diversity in *in vivo* “Brainbow”-like systems for which such database is difficult to construct; for example, plasmids of various versions of the Brainbow construct are available for *in vitro* testing. Our clonal assignment algorithm also facilitates the transfer of our strategy to *in vivo* “Brainbow”-like system; we demonstrated that, even with incomplete chromatic landscapes and unknown M_s_, the algorithm reports clonal frequencies comparable to those from FACS analysis. While we were consistent with our choice of FPs and their expression promoter (SFFV) in this study, we recognized that they are significant determinants of clonal color properties. Clones with inferior relative clonal brightness were mostly “blue” due to the relatively low (extinction coefficient × quantum yield) of Cerulean compared to Venus and tdTomato^44^,^45^; other FP properties affecting clonal color include pK_a_, which can render clonal colors pH sensitive when pK_a_ values are dissimilar between expressing FPs, and the presence of immature fluorescent species that have been reported for RFPs^46^. Differences in integration sites can explain the inter-clonal variations in chromatic spread and chromatic stability. We noted that chromatic instability did not, in most cases, correspond to the silencing of a FP marker, which could be identified by monotonic decrease in fluorescent intensities over time and chromaticity mode shift towards autofluorescence (data not shown).

Diversity of clonal color codes is necessary for fluorescent clonal tracking. We expanded our yield of distinguishable clonal colors in our candidate clonal pool during the generation of MelaChromas, from which our clonal founder cells would be drawn. We learned that there was an optimal dosage for generating the most color diverse MelaChromas and higher dosages did not yield more colors. We propose that toxicity of FPs at high expression levels was likely cause of reduced color diversity at high lentiviral transduction dosages and should be taken into consideration whenever color generation by combinatorial FP expression is implemented. Also, we developed a combinatorial gating scheme for single clonal founder cell selection that reduced the number of clones required to capture the available clonal colors, including those present in low frequencies, in the candidate clonal pool. While our clonal founder gating scheme was designed for *in vitro* use, its underlying principle extends to *in vivo* and *ex vivo* scenarios in which the spatial location of cells are used as clonal co-identifier, namely when cells of similar colors and in close proximity are assumed to share a common clonal origin. This assumption not only requires local presence of clonal founders in diverse color codes, but also that they are present at similar frequencies. The ability to verify the presence of diverse color codes once again requires knowledge of clonal colors, especially their size relative to the size of the color space.

We chose ct-MelaChroma’s fifteen participant clones exclusively from our candidate pool of 150 3FP+ clones. We can increase the number of trackable clones by including 1FP+ and 2FP+ clones. We can also introduce new clones to our pool. Relaxing the candidate clonal pool’s selection criteria (inclusion of outliers for chromatic stability, lowering b* and the 97.5% percentile cutoff for relative clonal brightness) would also increase the size of the pool but at the cost of clonal assignment accuracy. Maximal numbers of participant clones is achieved via full chromaticity grid coverage with bright, chromatically stable clones of small chromatic spreads. Full coverage of the chromaticity grid requires a color diverse clonal source, as we have demonstrated with MelaChromas. Improvements in the fluorophores’ photophysical properties, incorporation and detection methods will lead to smaller clonal chromatic spreads.

In summary, we have demonstrated a strategy for creating RGB-coded, multi-clonal populations that is extendable with advancements in imaging technology, fluorophore design, and labeling technology including genetic engineering. Our strategy, which allows rigorous and objective clonal identification of each cell by its RGB color-coding, is as follows: 1) create a diversely color-coded, multi-clonal population and establish a consistent color space that accommodates all RGB color codes; 2) in the established color space, triage individual clones suitable for clonal tracking by relative clonal brightness and chromatic stability; and 3) identify subsets of chromatically distinguishable clones for physical pooling based on clonal chromatic mode and spread. RGB color-codes can originate from the combinatorial expression of any long-term endogenous or exogenous cell labeling fluorophores. R, G, B intensity information can be collected from different imaging modalities. With sufficient computational power, our algorithms can accommodate large numbers of participant clones. Our clonal color metrics will also lay the foundation for building mathematical models that truly mimic clonal color behavior. We will be able to, for example, generate statistically robust functions describing the chromatic mode and chromatic spread for *each* candidate clone, which will streamline intra- and inter-clonal comparisons and facilitate the implementation of complex clonal assignment rules, cumulating to high assignment accuracy. Of note, our methods of clonal color and clonal composition analysis are not restricted to three-fluorophore systems. It can be expanded into *n*-dimension space with each dimension representing a fluorophore. Our strategy is also compatible with alternate clonal assignment methods. Mathematical models of clonal color behavior can be applied for clustering analysis. While our strategy does not require spatial location or morphological information, inclusion of additional spatial/morphological information will reduce the uncertainties and errors associated with color-based clonal assignment. Meanwhile, our strategy retains the ability to assign in sparsely-populated or highly dynamic environments, such as those in minimal residual disease setting. Integration of different clonal assignment methods shall lead to highly accurate and efficient multi-clonal tracking studies.

## Methods

### Production and titer calculation for LeGO lentivirus

Lentiviral Gene Ontology (LeGO) vectors LeGO-Cer2, LeGO-V2 and LeGO-T2 were generous gifts from Drs. Kristoffer Riecken (né Weber) and Boris Fehse at the University Medical Center Hamburg-Eppendorf. Plasmid replications were performed via transformation of NEB 5-alpha competent E. Coli (New England Biolabs, Ipswich, MA) and DNA purification with the QIAfilter Plasmid Midi Kit (Qiagen, Valencia, CA), according to manufacturer’s instructions.

Lentivirus generation and titer calculation followed previously published protocol^38^, scaled up 2.75×. Briefly, 1.375E7 HEK-293T cells were seeded in a T175 flask for an 8-hour transfection, for which 27.5 μg LeGO (Cer2, V2 or T2), 27.5 μg pMDLg/RRE, 13.75 μg rPSV-Rev and 5.50 μg pHCMV-VSV-G plasmids were added. Cells were subsequently filter-removed with 100 μm nylon mesh cell strainer and 0.45 μm PVDF syringe filters. For titer determination, HEK-293 T cells were seeded in 24-well plates at 5E4 cells per well, and transduced for 13 hours with 0.1, 0.5, 1.0, 5.0, 10.0, or 100 μL of viral supernatant. Three wells were transduced per supernatant volume. Percent fluorescent cells were determined three days later with flow cytometry. Calcium phosphate transfection kit was purchased from Sigma-Aldrich.

### Melanoma Cell Line

A375 human amelanotic melanoma cell line (ATCC) was cultured in phenol-red free DMEM supplemented with 10% fetal bovine serum, 2.6 g/L sodium bicarbonate, 3.5 g/L glucose (for 4.5 g/L total concentration) and 50 U/mL penicillin-streptomycin, and incubated in 37 °C, 5% CO_2_ humidified atmosphere. Cellstripper (non-enzymatic cell dissociation solution; Corning Inc.) was used for cell harvests.

### Generation of fluorescent protein expressing, multi-clonal human melanoma (MelaChroma)

Multi-clonal A375 populations expressing diverse fluorescent colors were named “MelaChromas”. MelaChomas, along with A375-Cerulean, A375-Venus and A375-tdTomato, were generated with minor modifications from previously published protocol^38^. A375 cells were seeded in 6-well plates, at 5E4 cells in 2.4 mL culture media per well. Two hours later, 8 μg/mL polybrene was introduced with lentiviral particles carrying LeGO-Cer2, LeGO-V2 and/or LeGO-T2, at dosages measured in multiplicity of infection (MOI). Six MOIs (0.35, 0.70, 1.4, 2.8, 4.9, and 7.0) were tested, two wells per MOI. MelaChromas were given all three lentiviruses at equal MOIs. A375-Cerulean, A375-Venus and A375-tdTomato were transduced, respectively, with only LeGO-Cer2, LeGO-V2 or LeGO-T2, each at the same MOIs given to MelaChromas. Cells were subsequently incubated for 16 hours, after which transduction was terminated by culture media change. Three days later, fluorescent protein expression was determined for each A375 population by flow cytometry.

### Creation and maintenance of 3FP- and 1FP- expressing MelaChroma clones

On Day 5 and Day 10 after MelaChroma generation, single cells were sorted into 24-well plates by FACS using a combinatorial RGB intensity gating scheme. Each well was pre-filled with 0.4 mL culture media supplemented with 25 mM HEPES. MelaChromas of MOI = 0.7, 2.8 and 4.9 were used as cell sources. For each MelaChroma, R/G/B channel gain settings were adjusted such that its fluorescence signals spanned the full intensity range of 0–65536 (16-bit) for fluorescence microscopy and 0–1E5 for flow cytometry. A total of 504 cells that expressed three FPs were plated for clonal expansion.

Wells were inspected weekly with a light microscope. Once a clone exhibited three dimensional growth, its cells were harvested and re-plated without sub-culturing. Maintenance of the clone then followed the protocol for A375 cell line.

A375-1FP clonal founder cells were similarly isolated from A375-Cerulean, A375-Venus and A375 tdTomato (MOI = 7.0) and expanded, on Day 32 after generation of the source populations.

### Clonally trackable MelaChroma generation and maintenance

Clonally trackable (ct)-MelaChroma was generated by mixing 1.2E6 cells from fifteen different clones. The cells (1.8E7 total) were incubated for 36 hours, harvested and seeded into six T75 flasks, at 2E6 cells each. Afterwards, the six flasks were sub-cultured at 1:8 twice every week, on identical schedules. Flow cytometry was performed on days of sub-culturing.

### Flow cytometry and confocal imaging

Flow cytometry was performed by the FACSAria installed with FACSDiva software (BD Biosciences). Cerulean fluorescent protein was excited at 405 nm and detected with a 475/20 bandpass filter. Venus was excited at 488 nm and detected with a 528/20 bandpass filter. tdTomato was excited at 561 nm and detected with a 582/15 bandpass filter. All optics settings, including voltage gains for R/G/B detection channels, were finalized on Day 26 after MelaChroma generation. Cells were harvested and re-suspended at 1–2E6 cells/mL DMEM (same formulation as culture media) supplemented with 1% FBS and 25 mM HEPES. A375-wild type (WT) autofluorescence was measured on all days of experiments.

For flow cytometric analysis of cell cycle, MelaChroma clones were pre-labeled with Vybrant DyeCycle Ruby stain (Invitrogen) according to manufacturer’s instructions. The dye was excited at 633 nm and detected with a 660/20 bandpass filter. “Spiked” clonal cells were pre-labeled with DRAQ5 (Abcam) according to manufacturer’s instructions. The dye was excited at 633 nm and detected with a 695/40 bandpass filter. Cerulean, Venus and tdTomato channel settings remained as above; fluorescence compensation was not necessary due to spectral separation.

Confocal imaging was performed on the Olympus FluoView FV1000 confocal microscope. Cerulean was excited with 458 nm argon laser line and detected between 475–495 nm. Venus was excited with 515 nm argon laser line and detected between 530–550 nm. tdTomato was excited with 559 nm laser diode and detected between 575–595 nm. 20×, 0.95 NA water immersion objective was used. Voltage gains were finalized on Day 32 after MelaChroma generation. Cells were pre-plated in Lab-Tek chambered cover glass (Thermo Scientific Nunc) or FluoroDish (WPI Inc.) for imaging. DMEM (same formulation as culture media) supplemented with 1% FBS and 25 mM HEPES was used as imaging media.

### Color data analysis

Raw RGB color data of singlet cells from flow cytometry were extracted using the software FlowJo (version 7.6; Tree Star) and imported by MATLAB (version 2011b; MathWorks) scripts created in-house for color analysis. MATLAB scripts for color analysis are available online at https://github.com/juwellwwu/ClnColorAnalysis.gitwith instructions and test datasets. Image registration was performed in the image processing software Fiji (ImageJA 1.45b) with the plugin bUnwarpJ downloadable at http://biocomp.cnb.csic.es//~iarganda/bUnwarpJ/47.

## Additional Information

**How to cite this article**: Wu, J. W. *et al*. Defining Clonal Color in Fluorescent Multi-Clonal Tracking. *Sci. Rep*. **6**, 24303; doi: 10.1038/srep24303 (2016).

## Supplementary Material

Supplementary Information
