# Supplementary material for: Defining Clonal Color in Fluorescent Multi-Clonal Tracking
Source: Sci Rep. 2016 Apr 13;6:24303. doi: 10.1038/srep24303 (PMC4829845; doi:10.1038/srep24303)
Supplement: Supplementary Information [file srep24303-s1.doc]

**Defining Clonal Color in Fluorescent Multi-Clonal Tracking**

Juwell W. Wu, Raphaël Turcotte, Clemens Alt, Judith M. Runnels, *Hensin Tsao, *Charles P. Lin

**SUPPLEMENTARY INFORMATION**

**SUPPLEMENTARY FIGURES**

**
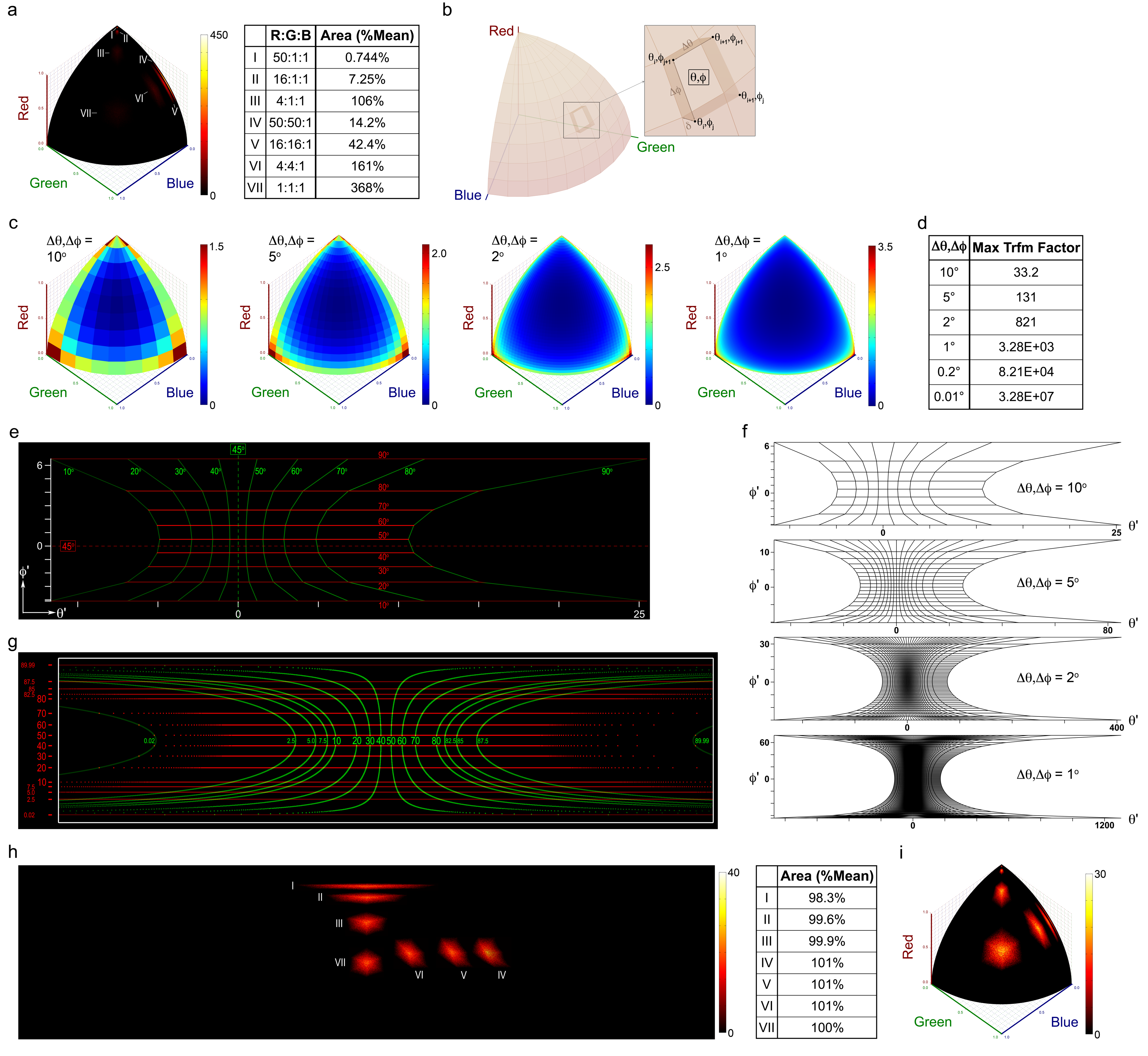
­­**

**Supplementary Figure S1: The Θ’-Φ’ grid.** The Θ’-Φ’ grid is a mathematically transformed chromaticity Θ-Φ grid that normalizes the chromaticity span for clones with similar RGB fluorescent intensity variations. It primarily serves to permit unbiased inter-clonal comparisons of clonal color properties. It also supplies the adjustment factors used for calculating adjusted cell counts, which enhance readability of spherical histograms (see **i**). **a**, Bias in inter-clonal comparisons of clonal color properties, in particular chromatic mode and chromatic stability, is caused by clones with identical RGB fluorescent intensity variations among their clonal cells spanning a different coverage area on the chromaticity grid. This coverage area is a function of the clones’ position on the grid. To illustrate this concept, we simulated seven “model clones”, each with 1E5 “clonal cells”. These clones had different R:G:B peak intensity ratios (*I_p,R_ :I_p,G_ :I_p,B_*) but identical intensity variation in each channel (*ΔI/I_p_*=*ΔI_R_/I_p,R_* =*ΔI_G_/I_p,G_* =*ΔI_B_/I_p,B_* =0.25). Span of these clones varied considerably on the spherical histogram, quantified as the percentage of the mean coverage area of the seven clones on the grid. Clones with disparate *I_p,R_ :I_p,G_ :I_p,B_* ratios (positioned near the edges and corners) occupied significantly less area than those with near-unity ratios (positioned near the center). Spherical histogram containing all seven clones was difficult to read as a result, as the smaller-spanning clones with disparate *I_p,R_ :I_p,G_ :I_p,B_* ratios carried correspondingly higher cell counts per chromaticity grid element that required cell count scales different from clones with near-unity ratios. The adjusted cell count scale, calculated based on the Θ’-Φ’ grid’s transformation factors, will circumvent this readability problem (see **i**). **b**, We created the Θ’-Φ’ grid to normalize the areal discrepancies observed in **a**. Each (Θ,Φ) coordinate, as the histogram bin center for (Θ±0.5ΔΘ,Φ±0.5ΔΦ), corresponds to a Cartesian RGB “shell” of infinitesimal thickness δ enclosing specific R, G, and B fluorescence intensity ranges. This shell volume and its enclosed range of RGB intensities vary with (Θ,Φ). Clones with a given RGB intensity variation therefore span a different number of histogram bins depending on the clone’s (Θ,Φ) coordinate. Based on this reasoning, we postulated that the shell volume at each (Θ,Φ) is inversely related to the transformation factors needed to normalize the areal discrepancy. We therefore resized each grid element on the Θ-Φ grid by assigning each Θ-Φ grid element a new area equal the inverse of the shell volume enclosed in (Θ±0.5ΔΘ,Φ±0.5ΔΦ) post-normalization (such that the smallest value=1). The Θ’-Φ’ grid was outcome after arranging the new (Θ’,Φ’) grid elements into grid form, rescaling the axes and setting Θ=Φ=45° as the new origin (see **e**-**g**). **c**, Transformation factors, or the new areas for each (Θ, Φ) grid element, in log10 scale plotted on the chromaticity grid. Grid element size ΔΘ, ΔΦ = 10, 5, 2, 1°. **d**, Maximum transformation factors for different Θ, Φ. All computations in this manuscript used ΔΘ, ΔΦ = 0.01° except for the calculation of adjusted cell counts in spherical histograms (ΔΘ, ΔΦ = 0.2°) (see **i**). **e**, Θ’-Φ’ grid, ΔΘ, ΔΦ = 10°. Locations of the original Θ (green) - Φ (red) grid lines show the arching of the Θ-Φ grid as the result of “stretching” the area of individual Θ-Φ grid elements. The 5° bins between 0°-10° grid lines were included in the calculations (the 0° gridlines were not plotted). **f**, Θ’-Φ’ grids, ΔΘ, ΔΦ = 10, 5, 2, 1°, Θ’, Φ’ axes in 1:1 aspect ratio. The grids share a “candlestick” appearance, but with increasingly wide “ledges” at high and low Θ, Φ values. **g,** Θ’-Φ’ grids shown in this manuscript are partial grids (ΔΘ, ΔΦ = 0.01°) for visual clarity. Locations of the original Θ (green) - Φ (red) grid lines are shown. **h**, Histogram of the seven “model clones” from **a** on the partial Θ’-Φ’ grid shown in **g.** The table lists their span in the Θ’-Φ’ grid, again compared to the mean coverage size of the seven clones, showing that clones with identical RGB intensity variation (*ΔI/I_p_*) had near-uniform span on the Θ’-Φ’ grid. This feature of the Θ’-Φ’ grid allows unbiased inter-clonal comparison of clonal color properties. **i**, Adjusted cell counts, calculated using transformation factors, improve readability of spherical histograms. Adjusted cell counts are calculated by dividing the binned cell counts of each Θ-Φ grid element (ΔΘ, ΔΦ = 0.2°) by the transformation factor of the same element. This adjustment serves purely illustrative purposes: conceptually, it is equivalent to distributing the cell counts in each Θ-Φ grid element into a transformation-factor-value number of sub-elements. The spherical histogram remains visually accurate as long as individual Θ-Φ grid elements (ΔΘ, ΔΦ = 0.2°) are single point or single pixel-sized in print. The spans of the seven clones in this spherical histogram plotted with adjusted cell counts are the same as in **a**, and highly readable.


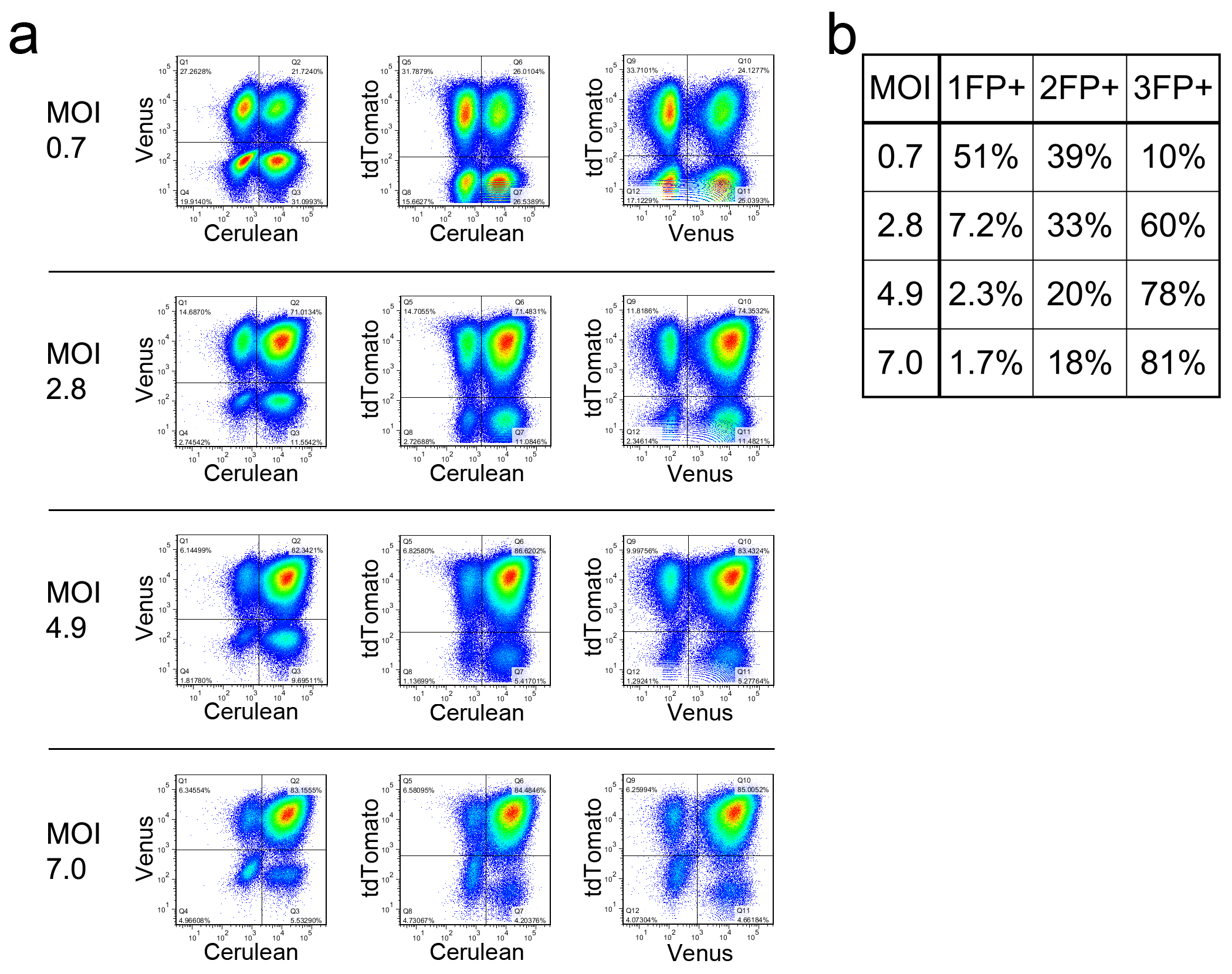


**Supplementary Figure S2: Color properties of MelaChroma. a**, Flow cytometry plots of MelaChromas, 32 days after lentiviral transduction, showing enrichment of 3FP+ expressing cells with increasing MOI. Cells without FP expression were removed. >1.72E5 cells were analyzed by flow cytometry per population. **b**, Percentages of MelaChroma cells expressing 1FP, 2FPs and 3FPs.


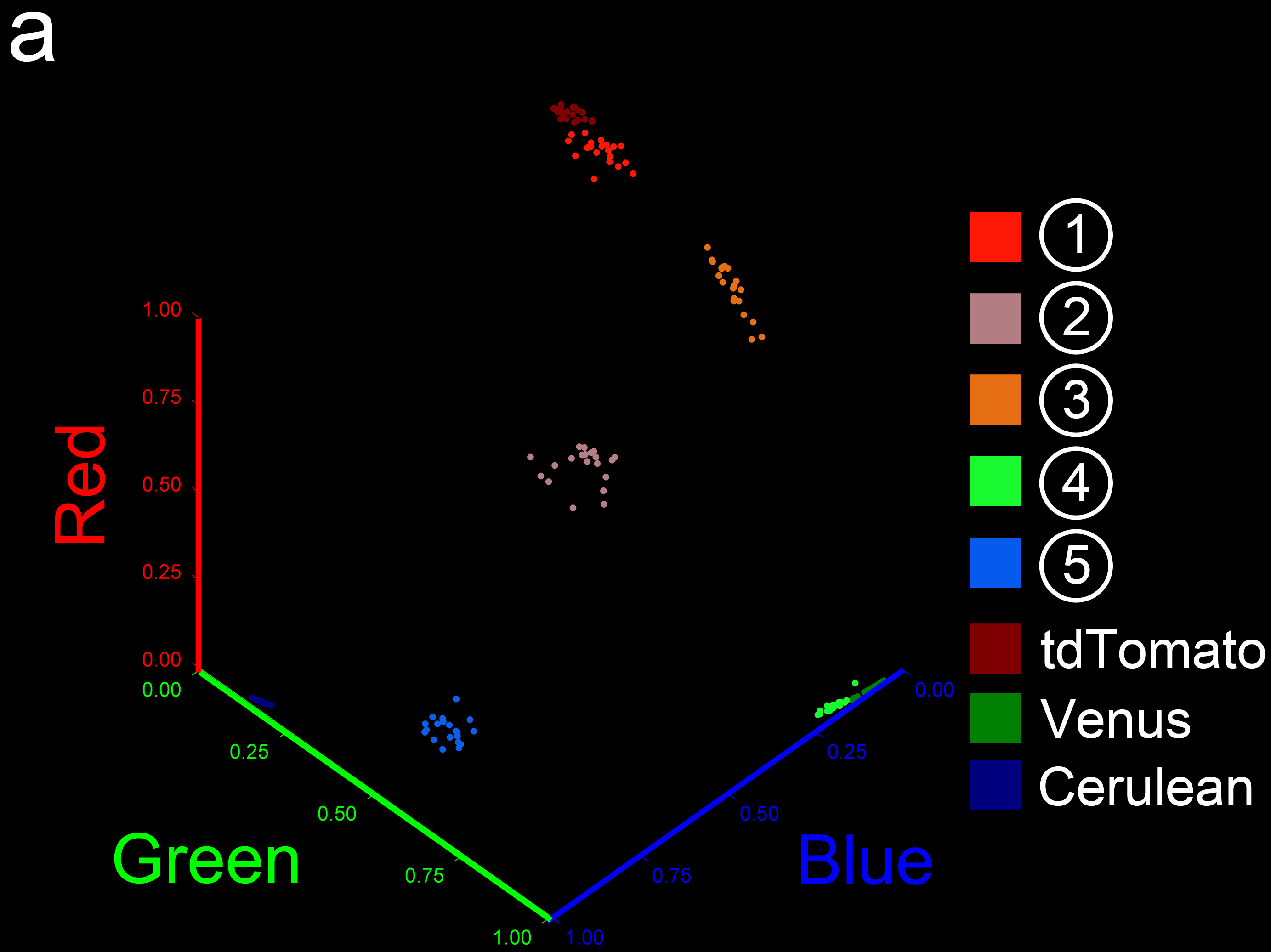


**Supplementary Figure S3: Cell color analysis of confocal images of MelaChroma clones. a**, Spherical scatter plot of randomly selected cells from the confocal image of MelaChroma clones (Fig. 2**a**). Each cell was defined as a region-of-interest in Fiji and its mean RGB value was analyzed. 20 cells were analyzed for each clone.


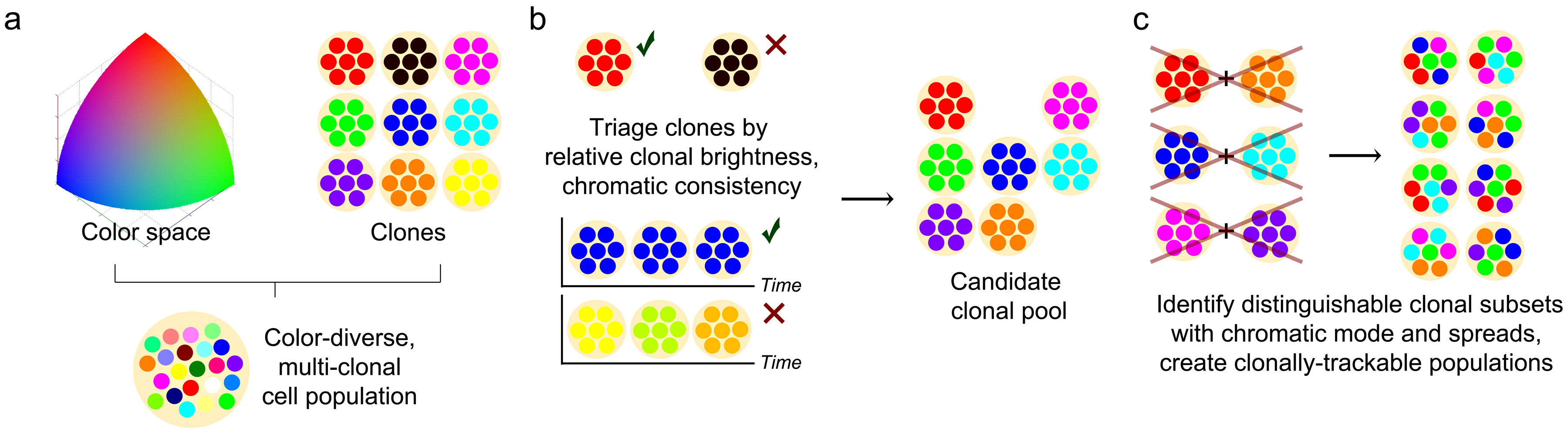


**Supplementary Figure S4: Use of clonal color metrics in fluorescent clonal tracking. a**, Using clonal color metrics requires a well-defined and consistent a color space. The color space is established with a color diverse, multi-clonal population, which can also act as the source of the clones. **b,** Candidate clones for long term fluorescent clonal tracking are qualified by individual clonal color criteria, namely, relative clonal brightness and chromatic stability. **c**, Distinguishable subsets of clones from the candidate pool are identified by comparing their chromatic mode and chromatic spreads. Multi-clonal populations generated by pooling these clones are clonally trackable.


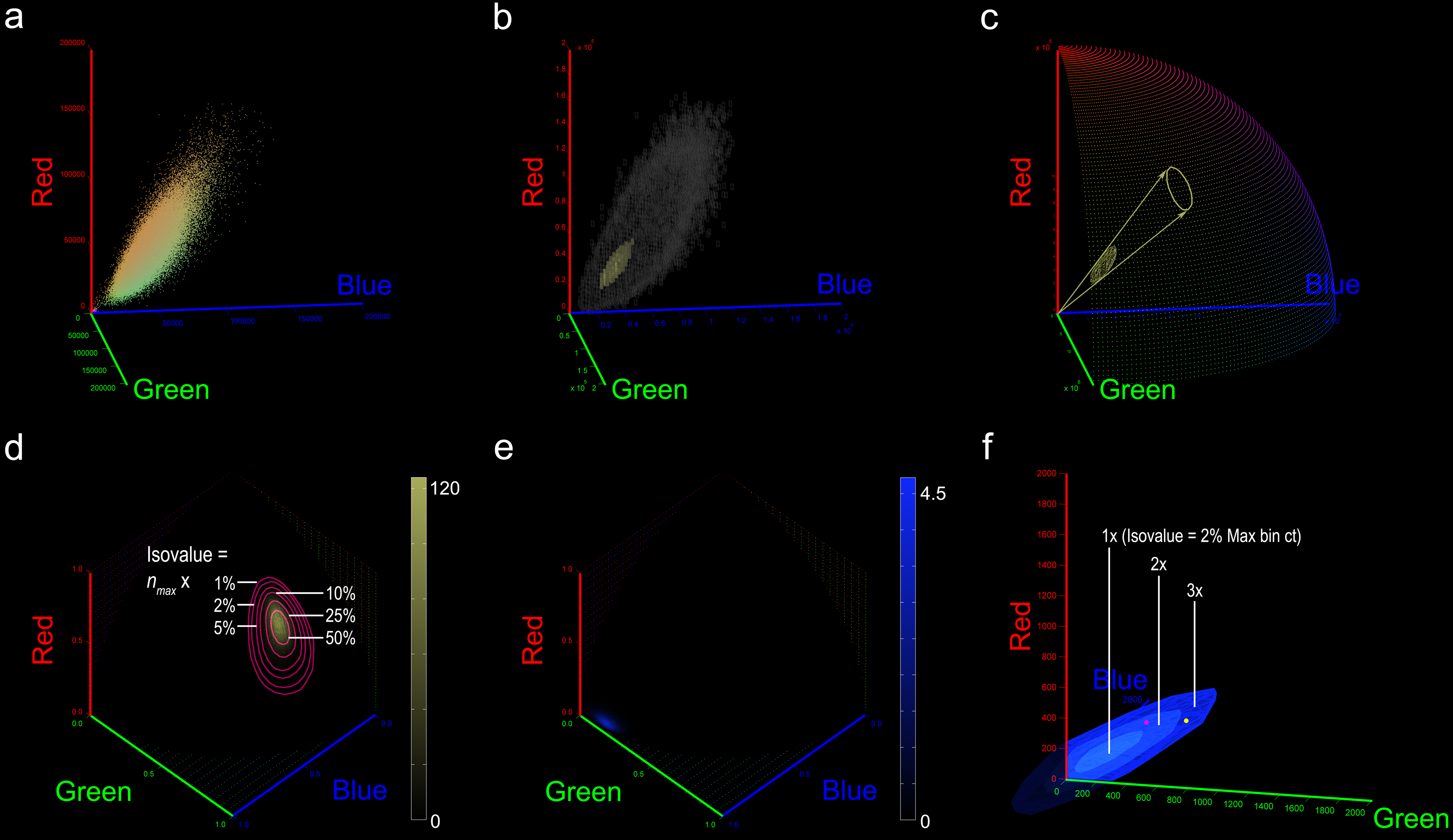


**Supplementary Figure S5: Calculating chromatic mode and chromatic spreads; autofluorescence as a unit of relative cell brightness. a**, Clonal chromatic mode and chromatic spreads are computed from 3D histogram binning of clonal cell colors. A “comet-shaped” RGB cell color cloud was characteristic for most MelaChroma clones. **b**, The chromatic mode is the value-normalized RGB coordinate of the 3D histogram bin center with the highest clonal cell count (*n_max_*). Chromatic spreads are 2D projections of isosurfaces drawn at user-specified fractions of *n_max_*; a *% n_max_* isosurface is expected to enclose (*1- %n_max_*) of clonal cells within its boundary. 3D histogram binning (100 bins per axis) of the RGB cell color cloud in **a** is shown. The bins enclosed by the 50% isosurface are colored in green. **c**, Chromatic spreads, as 2D projections of isosurfaces onto the chromaticity grid, are contour lines around the chromatic mode. The 50% isosurface from **b** and its corresponding chromatic spread are shown. **d**, In this manuscript, six arbitrary %*n_max_* values were chosen for chromatic spreads (%*n_max_*=50%, 25%, 10%, 5%, 2%, 1%). When overlaid on the clone’s spherical histogram, chromatic spreads co-localized with the region of high cell count. **e**, Autofluorescence (AF), like clonal colors, can be characterized with chromatic mode and chromatic spreads. Spherical histogram of 6.7E4 non-FP expressing A375 (A375-WT) cells is shown. **f**, We defined relative cell brightness as a cell’s fluorescence in units of xAF (Multiples of Autofluorescence), and 1 xAF as the 2% isosurface of A375-WT. We reason that as the 2% isosurface, enclosing 98% of A375-WT cells, describe AF’s maximum possible contribution to each cell’s RGB fluorescence, relative cell brightness becomes a measure of the relative contribution of fluorescent protein versus AF towards the cell’s total fluorescence. The relative cell brightness value is determined geometrically. A cell of relative brightness 3 xAF (yellow dot), for example, is inside the 1 xAF volume magnified three times in size while centered at AF’s chromatic mode. The magenta cell, meanwhile, has a brightness of 2 xAF. Relative clonal brightness is the fraction of clonal cells that surpass a sufficiently high relative cell brightness benchmark value *b^*^*.


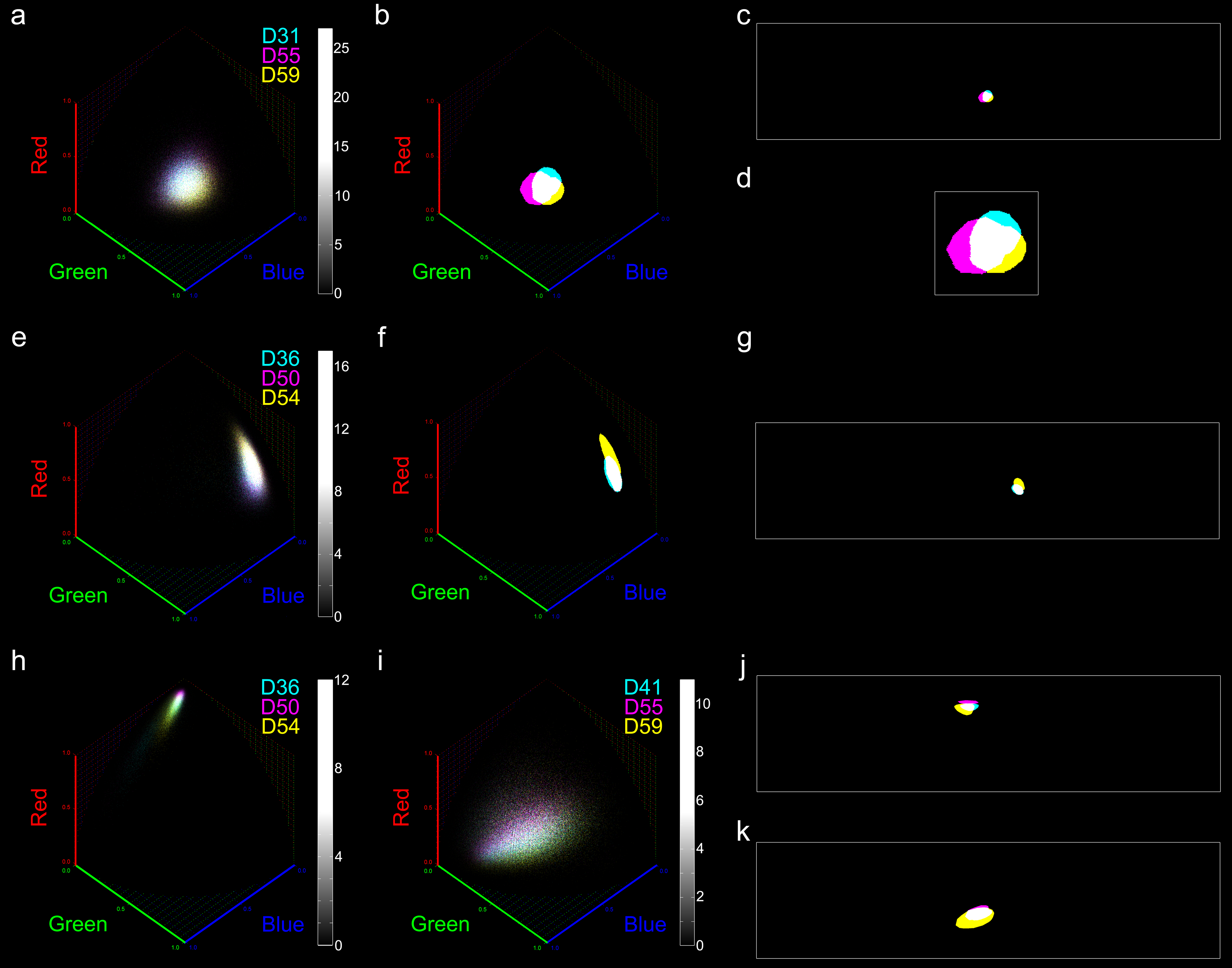


**Supplementary Figure S6: Calculating chromatic stability. a**, Chromatic stability measures the clonal chromaticity shift over time. These shifts can be observed in the overlay of spherical histograms of a clone’s color data. Cyan, magenta and yellow represent three different days of clonal color data collection (D0=day of clonal founder cell sorting). Comparing the shifts between different clones is nonetheless difficult and the number of time points allowed is also restricted. **b**, Chromatic stability enumerates the shifts observed in **a**. Plotting chromatic spreads (50% isosurface) produces well-defined boundaries for measuring the shifts. **c**, The same plot in **b**, re-plotted on the partial Θ’-Φ’ grid (Supplementary Fig. S1**g**). The mathematical transformation is necessary as chromatic spreads are sensitive to the clones’ chromatic position. **d**, Magnified view of the chromatic spreads occupied area in **c**. Chromatic stability was calculated by the formula *{(UN-MIN)/#Meas}*/*MIN*. *MIN* is the smallest single-time-point chromatic spread area of the clone in the Θ’-Φ’ grid (D31 for this clone). *UN* is the union area of chromatic spreads of all time points in the Θ’-Φ’ grid, i.e. the total colored area. *#Meas* is the number of time points. Chromatic stability of this clone is {(6832-3400)/3}/3400=0.336. **e**,**f**,**g**, Another clone with chromatic stability of 0.336, which is difficult to identify visually because of the clone’s different chromatic position. **h**,**i**, Another example of similar chromatic consistencies (1.007 for clone **h**, 1.004 for clone **i**) that are difficult to identify by spherical histograms. Clone **h** appeared smaller and more chromatically consistent due to its chromatic position and smaller chromatic spread size. **j**,**k**, Chromatic spreads (50% isosurface) of clone **h**(**j**), **i**(**k**) in the Θ’-Φ’ grid. Flow cytometry data of 5E4 cells were plotted for each time point in **a,e,h,i**.


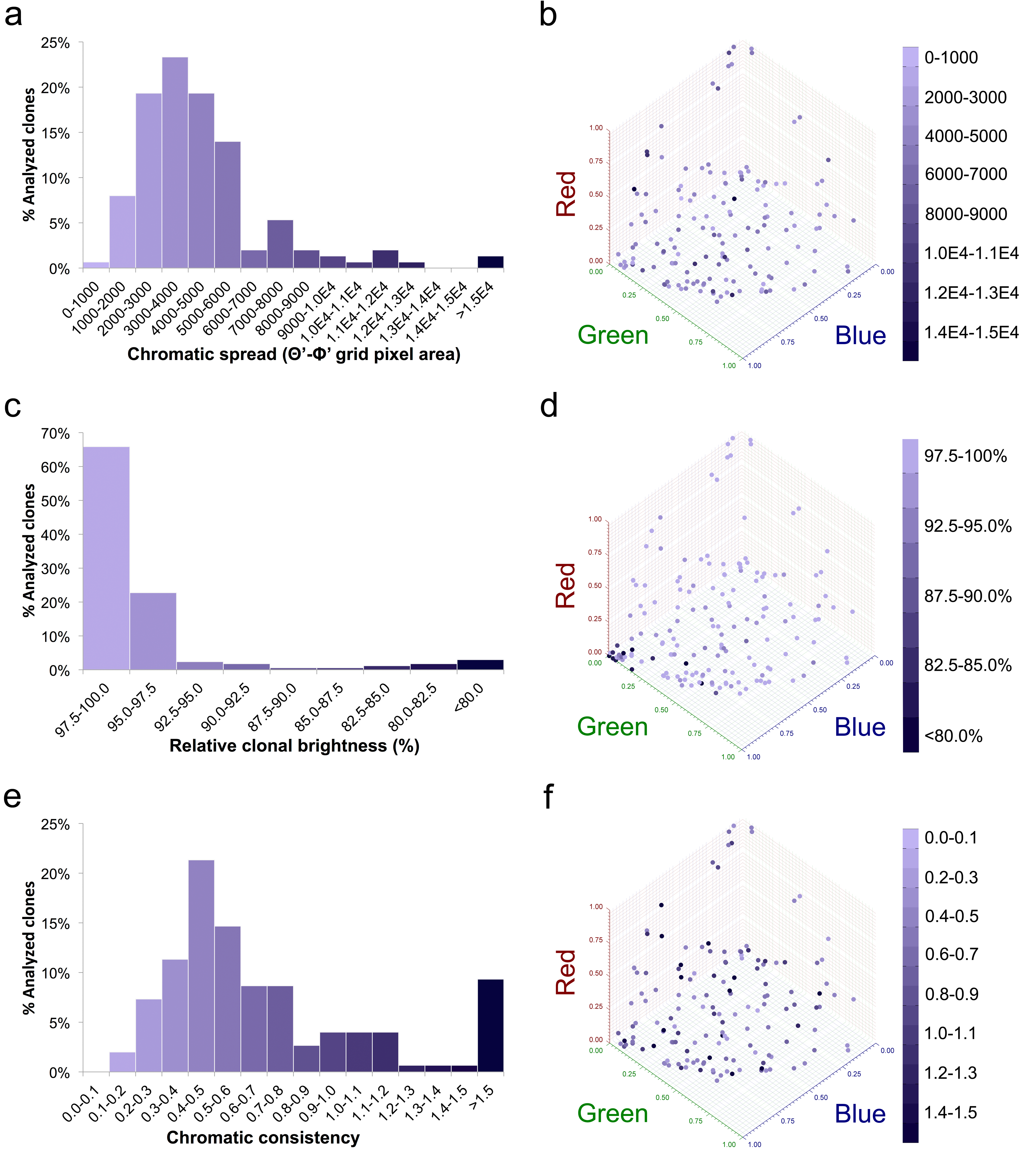


**Supplementary Figure S7: Chromatic spread, relative clonal brightness and chromatic stability of MelaChroma clones. a**, Chromatic spread (50% isosurface) of 150 MelaChroma clones (out of the 256 total) that had undergone n≥3 independent flow cytometry color measurements and their chromatic spreads contained within the partial Θ’-Φ’ grid (Supplementary Fig. S1**g**). Low chromatic spread values are desired. For reference, the total pixel area covered by the region [Θ’ Φ’=10°-80°] is 2.488E5. **b**, Spherical scatter plot showing the chromatic mode of analyzed clones, color-coded by their chromatic spread grouping in **a**. No chromatic clustering was observed among clones with small (light purple) or large (dark purple) chromatic spreads. **c**, Relative clonal brightness of 167 MelaChroma clones (out of the 256 total) that had undergone n≥3 independent flow cytometry color measurements. The benchmark *b^*^* value was set at 20 xAF, corresponding to <5% autofluorescence contribution to each cell’s total fluorescence. High values are desired. Ideal value is one. **d**, Spherical scatter plot showing the chromatic mode of analyzed clones, color-coded by their relative clonal brightness grouping described in **c**. Clones with poor relative clonal brightness (dark purple) congregated at the blue corner of the chromaticity grid. **e**, Chromatic stability of 150 MelaChroma clones (out of the 256 total) that had undergone n≥3 independent flow cytometry color measurements and their chromatic spreads contained within the partial Θ’-Φ’ grid (Supplementary Fig. S1**g**). Low values are desired. Ideal value is zero. **f**, Spherical scatter plot showing the chromatic mode of analyzed clones, color-coded by their chromatic spread grouping in **e**. No chromatic clustering was observed among clones of high (light purple) or low (dark purple) chromatic stability. >1.4E5 cells were analyzed by flow cytometry for all clones in **a**-**f**.


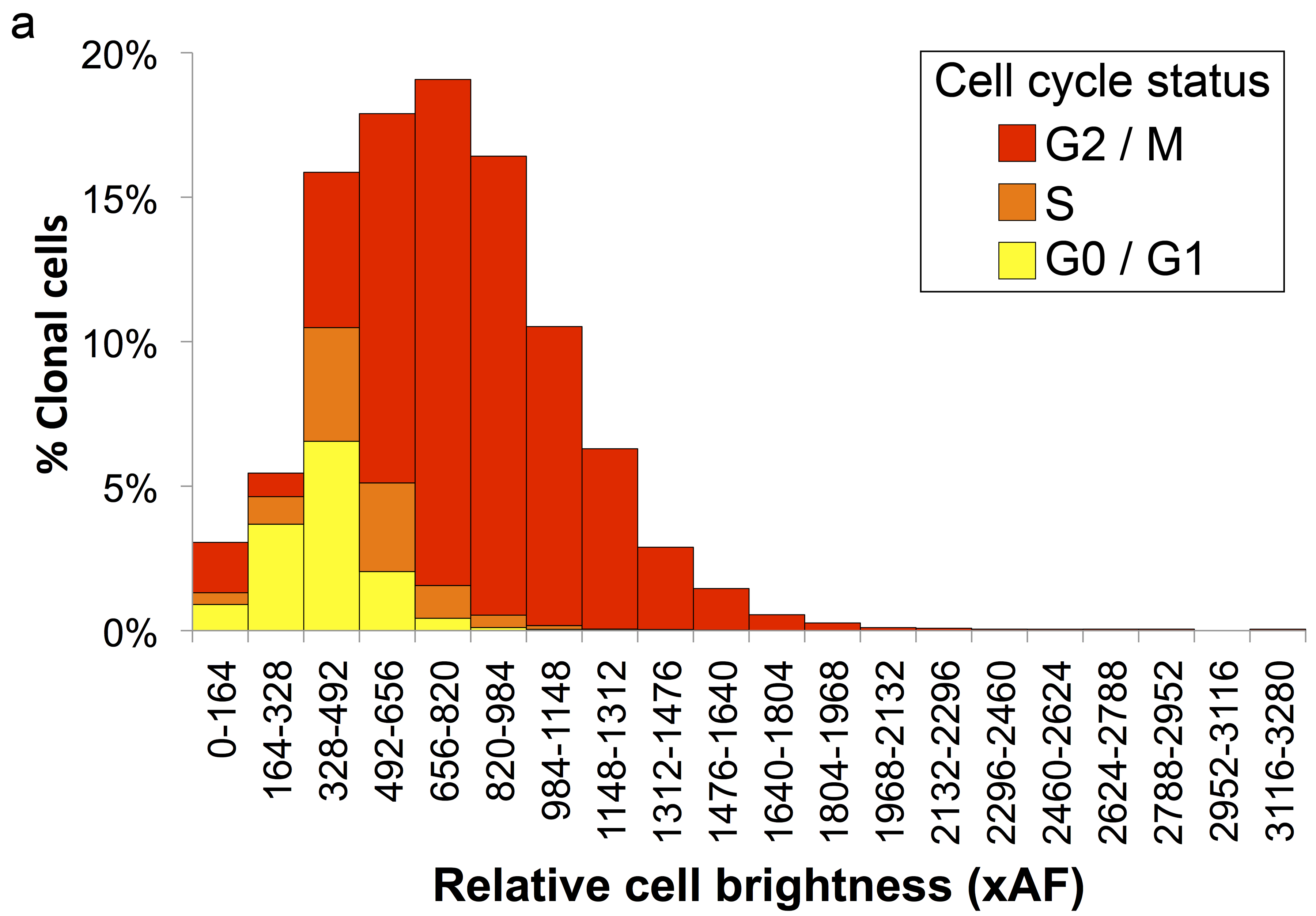


**Supplementary Figure S8: Relative cell brightness in a clone and its dependence on cell cycle status.** **a**, Relative cell brightness in a typical clone. Cells in different phases of the cell cycle were identified using Vybrant DyeCycle Ruby stain. 13.8% cells were in G0/G1 phase, 10.2% in S phase and 76.0% in G2/M phase. 4.82E4 cells were analyzed by flow cytometry.


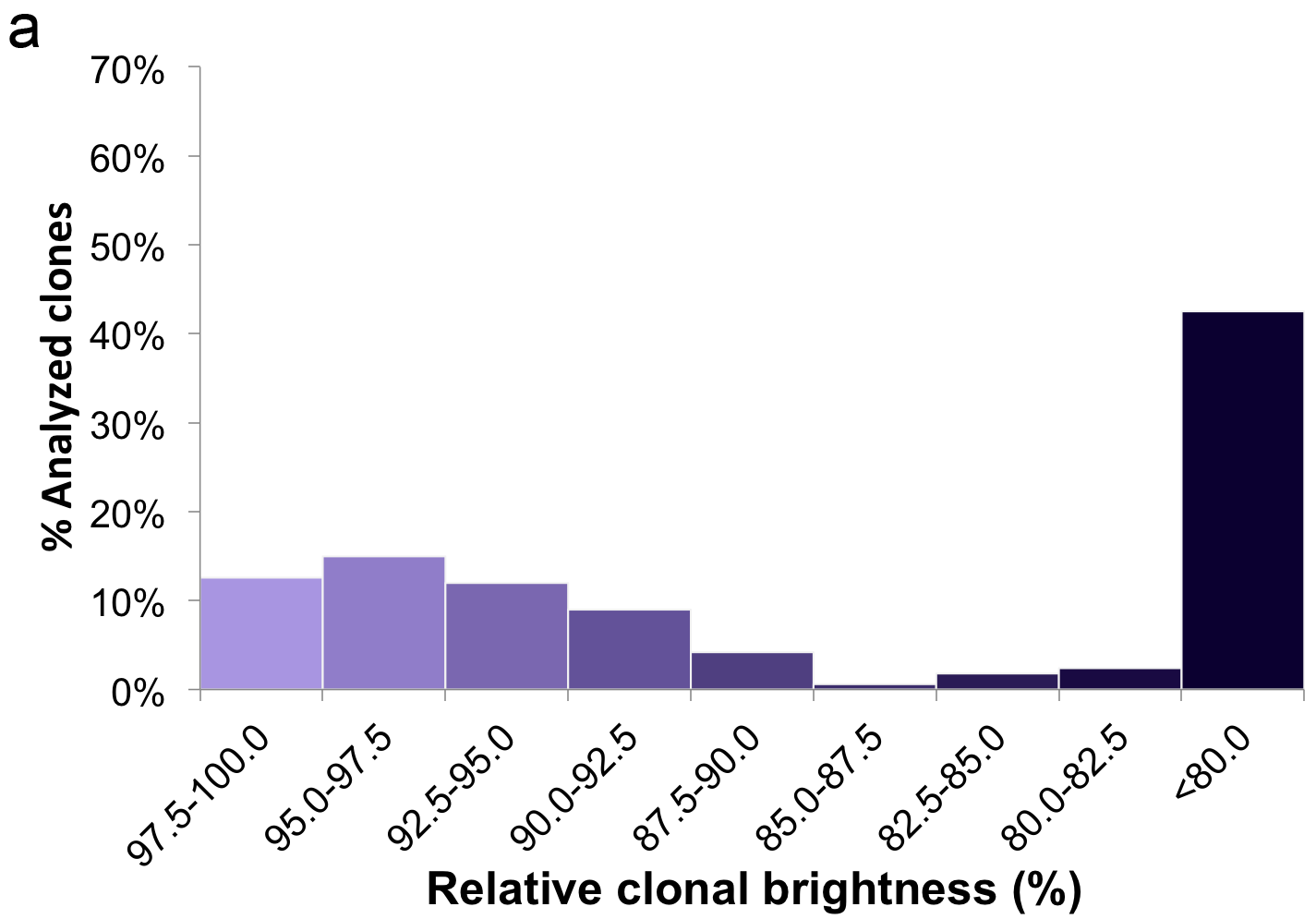


**Supplementary Figure S9: Effect of benchmark *b** value on the size of candidate clonal pool.** Relative clonal brightness of 167 MelaChroma clones (out of the 256 total) that had undergone n≥3 independent flow cytometry color measurements. The benchmark *b^*^* value was set at 100 xAF, corresponding to <1% autofluorescence contribution to each cell’s total fluorescence. 12.6% of the clones passed the 97.5 percentile relative clonal brightness criteria, compared to 65.9% for *b*=20* (Supplementary Fig. S7**c**).


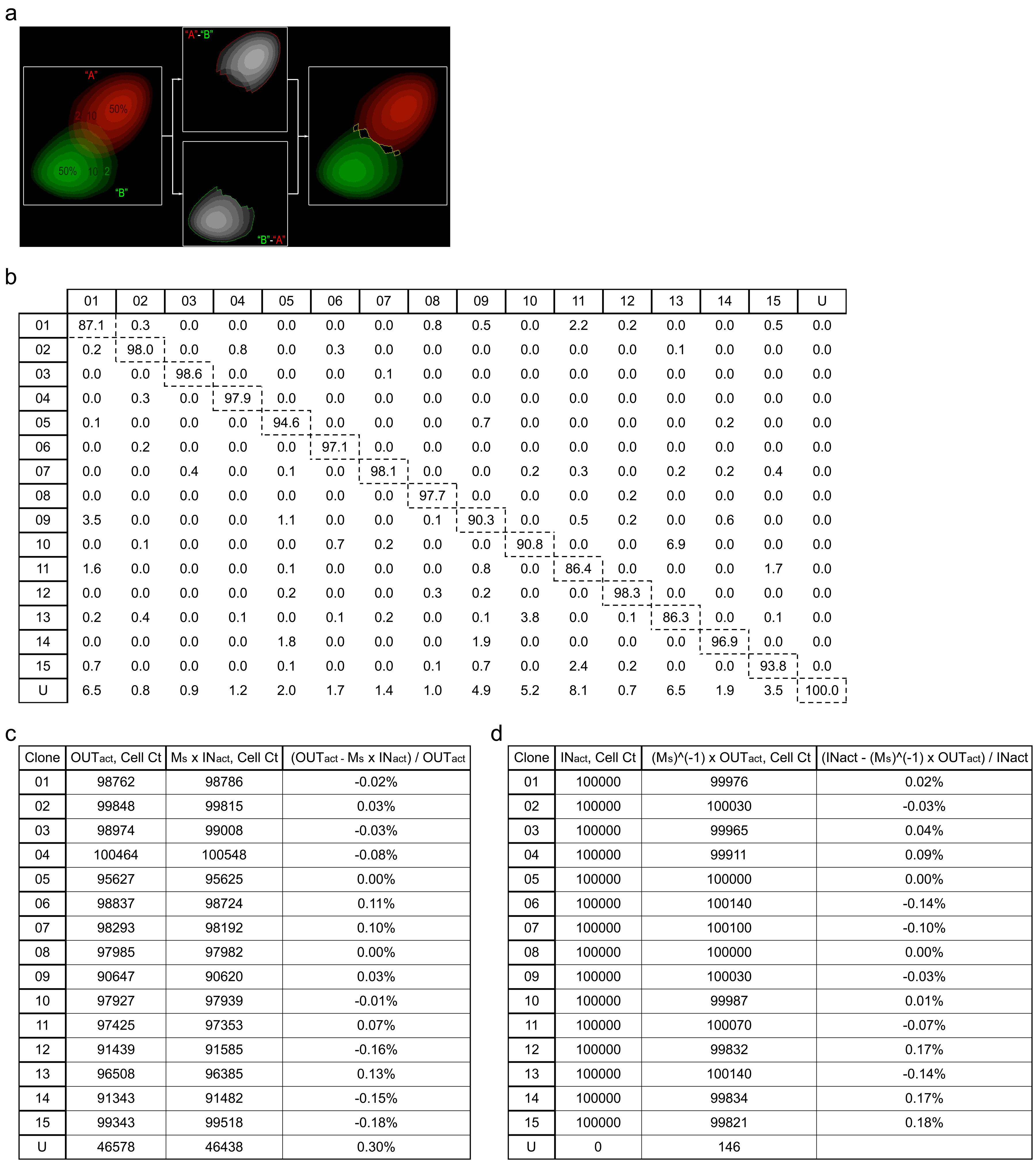


**Supplementary Figure S10: Clonal assignment rules for overlapping clones; validating the chromatic landscape and creating the spillover matrix. a**, Inclusion of low %*n_max_* chromatic spreads for clonal identification promotes comprehensive clonal assignment, but often at the cost of having (Θ,Φ) coordinates mapped to multiple clones. Digital pooling of clonal color data identifies these difficult-to-assign coordinates as the ones enclosed by chromatic spreads of more than one clone. In this manuscript, we defined an illustration-friendly assignment rule for these coordinates as follows. Overlapping chromatic spread of two hypothetical clones (“A”, “B”) were painted red and green respectively, with numbers denoting %*n_max_* of the isosurfaces. Each (Θ,Φ) coordinate in the overlapping regions was assigned to the clone with higher occupation probability, i.e., with higher %*n_max_* chromatic spread. Graphically, Clone “A” was allocated the positive intensity areas after the green channel was subtracted from the red channel (upper image) and Clone “B”, vice versa (lower image). The red and green lines bordered the two revised clonal boundaries, respectively. (Θ,Φ) coordinates in regions outlined in yellow, within the same %*n_max_* chromatic spread for Clone “A” and Clone “B”, were left unassigned. **b**. Validation of the chromatic landscape for clonal assignment also creates the spillover matrix M_s_. We clonally assigned 1E5 cells from each of ct-MelaChroma’s fifteen participant clones (column labels 01-15) using ct-MelaChroma’s chromatic landscape (Fig. 3**c**). The assignment outcome, in % frequency, was listed by the clonal identity assigned (row labels 01-15; “U” stands for “Unassigned”). The percentage of cells correctly assigned for each clone, in dotted boxes, was >86% for all MelaChroma clones. The percentage of clonal cells assigned to a wrong clone = 100% - (correctly assigned %) - (Unassigned %). This arrangement of validation results formed the spillover matrix M_s_ for ct-MelaChroma, specific both to the participant color clonal properties and clonal assignment rules described in **a**. **c**, Utility of the spillover matrix M_s_ is demonstrated by clonally assigning a “virtual” multi-clonal cell population with known clonal composition. We digitally pooled the color data from ct-MelaChroma’s fifteen participant clones, at 1E5 cells per clone, and created a column vector (IN_act_) of cell counts from each clone (row 1 to 15 for Clone 01 to Clone 15 =1E5; row 16 for Unassigned=0) to describe this population. The matrix multiplication M_s_ x IN_act_ closely approximated the assignment outcome OUT_act_, hence M_s_ succinctly described the error associated with our assignment approach. **d**. Conversely, (M_s_ )^-1^ x OUT_act_ estimated the actual clonal composition of our multi-clonal population, which was IN_act_.


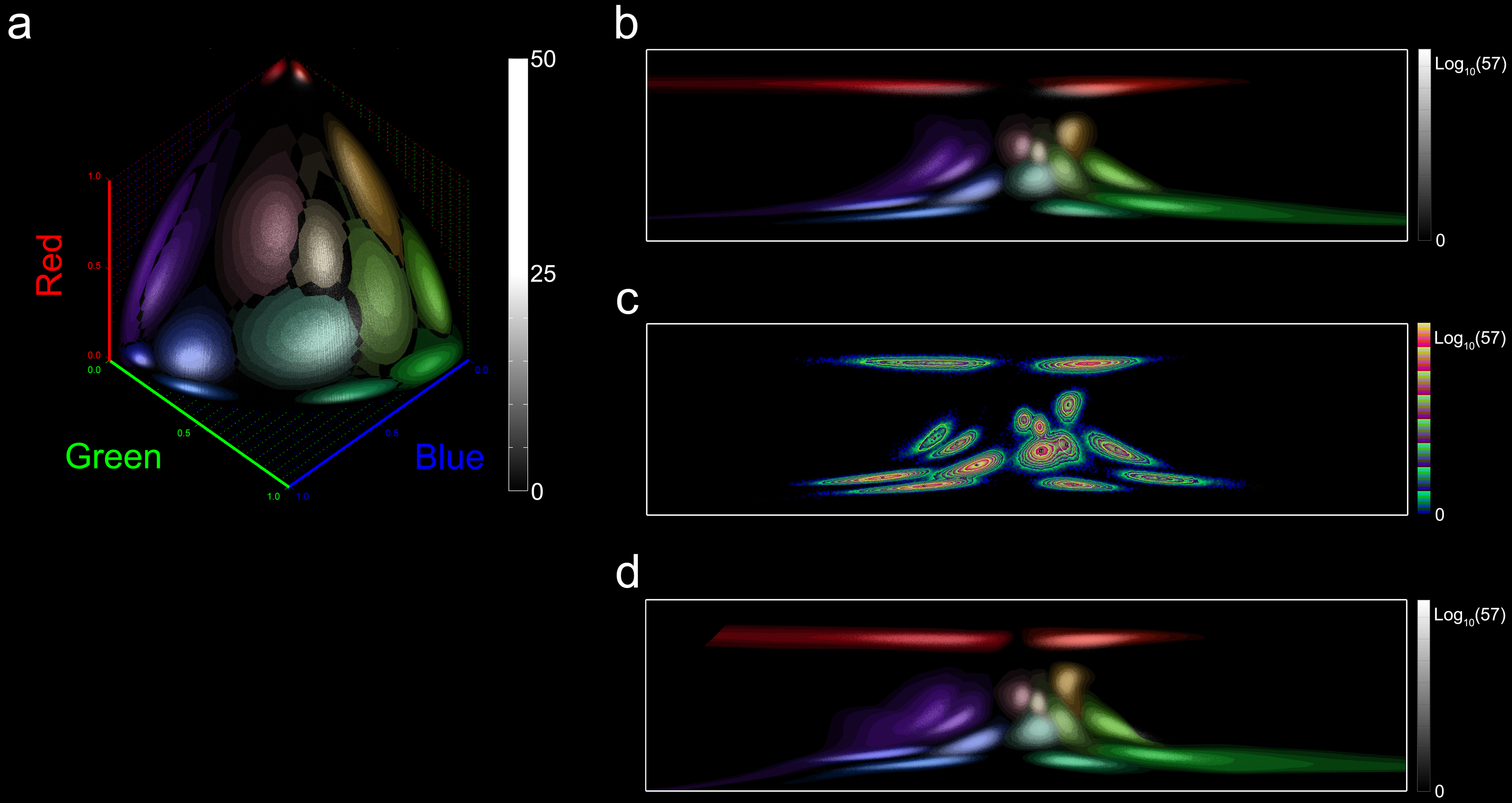
­­­­

**Supplementary Figure S11: Correction of chromaticity mismatches due to non-ideal clonal chromatic stability. a**, Non-ideal clonal chromatic stability manifests as minor mismatches between ct-MelaChroma’s chromatic landscape and the high cell count regions in the actual multi-clonal population’s spherical histogram analyzed at different time points. In this manuscript, we used the open source ImageJ algorithm “bUnwarpJ”^1^ to correct these mismatches. **b,** As bUnwarpJ’s correction was feature-based, **a** was re-plotted on the Θ’-Φ’ grid such that clones near the corners and edges of the chromaticity grid would not be penalized due to their chromatic positions (Supplementary Fig. S1**a**). **c**, bUnwarpJ calculated the Θ’ and Φ’ values for each Θ’-Φ’ grid element that best registered all ct-MelaChroma’s fifteen participant clones, after each clone’s 50% chromaticity spread in the chromatic landscape (outlined in yellow) was manually aligned to its peak in ct-MelaChroma’s spherical histogram (LUT: *3-3-2RGB*). **d**, **b** after image registration, showing diminished chromaticity mismatches. The same plot re-plotted on chromaticity grid is shown in (Fig. 3**e**).


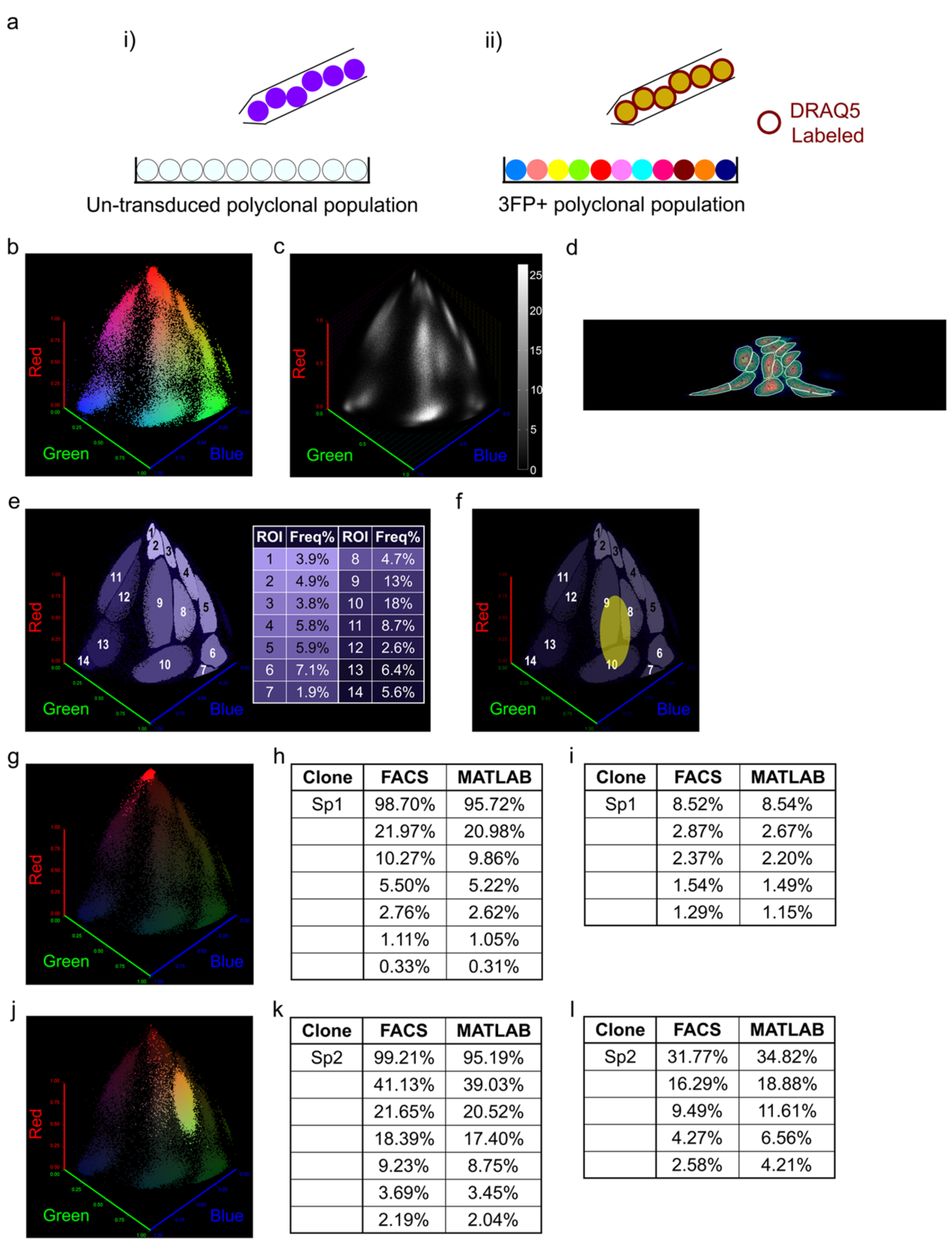


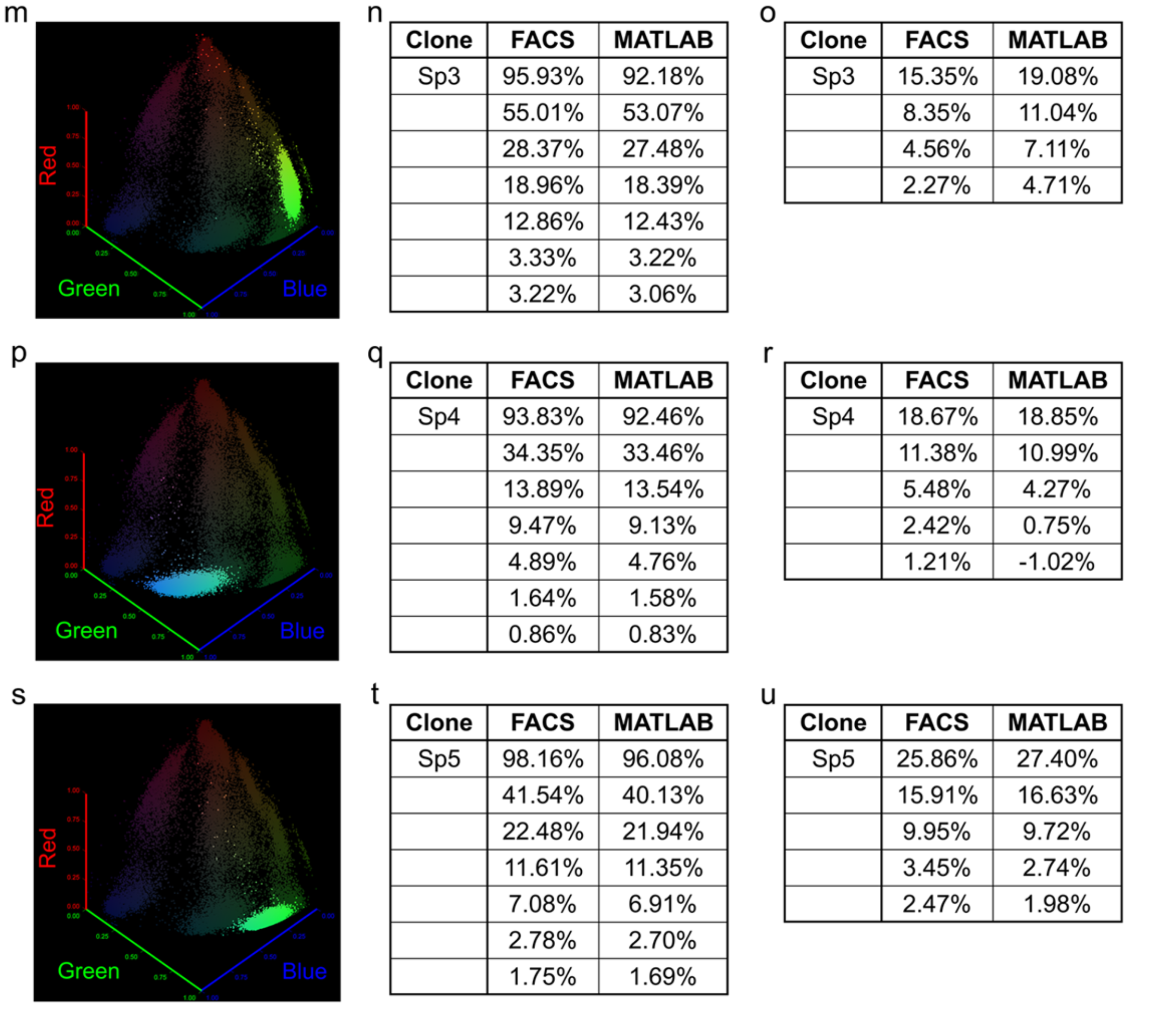


**Supplementary Figure S12: Clonal assignment with missing clonal color information. a**, Experimental scheme. We tested our clonal assignment algorithm’s ability to recover chromatically characterized clones “spiked” into an uncharacterized polyclonal background. Five different MelaChroma clones (Sp1-5) were each mixed with i) un-transduced and ii) 3FP+ polyclonal A375 populations at various spike levels *k%*. Chromatic mode and chromatic spread of SP1-5 were analyzed for clonal assignment. The polyclonal population was expanded from 120 3FP+ founder cells; its exact number of constituent clones and their chromatic properties were unknown. The nuclear dye DRAQ5 distinguished Sp1-5 cells from the polyclonal cells. **b**, Spherical scatter plot of the 3FP+ polyclonal population. ~1.2E6 cells are plotted**.** **c**, Spherical histogram of **b**. **d**, **c** re-plotted on the partial Θ’-Φ’ grid, cell count in Log_10_ scale. Setting the LUT to *3-3-2RGB* in Fiji shows fourteen distinguishable peaks, each outlined in white as a region-of-interest (ROI). **e**, Spherical scatter plot of the polyclonal population painted by each cell’s assigned ROI. Each ROI encloses a specific frequency of the 3FP+ polyclonal population, determined using the clonal assignment algorithm with each ROI designated as 1% chromatic spread input. **f**, Method of determining the spike level *k%*. *k* can be determined provided that at least one of polyclonal ROIs in **e** do not overlap with the chromatic spreads of the spiked clone. We illustrate this with an arbitrarily drawn yellow region that represents the known chromatic spread of a “spiked” clone. Using this yellow region as 1% chromatic spread input, our clonal assignment algorithm reports an assigned cell count value of *n_Sp_+n_ROI8_+n_ROI9_+n_ROI10_* for the spiked population, where *n_Sp_* is the cell count contribution from the “spiked” clone and *n_ROI8_,* *n_ROI9_,* *n_ROI10_* are the cell counts from the overlapping regions inside ROIs 8, 9 and 10. The values of *n_Sp_* and *k%* = (*n_Sp_* */ # singlet cells)* are extracted after estimating the value of *n_ROI8_+n_ROI9_+n_ROI10_*, performed as follows. First, we ran the clonal assignment algorithm on the un-spiked polyclonal population again using the yellow region as 1% chromatic spread input. This step provided the frequency of polyclonal cells enclosed in the overlapping regions inside ROIs 8, 9 and10 (*f_ROI8_+f_ROI9_+f_ROI10_*). Then, we approximated the polyclonal cell count in the “spiked” population, *n_Pc_*, by dividing the cell count assigned to any one of the non-overlapping ROIs (1-7, 11-14) by its corresponding frequency value in **e.** Finally, we calculated *n_ROI8_+n_ROI9_+n_ROI10_* by multiplying *n_Pc_* and (*f_ROI8_+f_ROI9_+f_ROI10_*). **g**, Spherical scatter plot of clone Sp1 (~1.2E4 cells) overlaid on the spherical scatter plot of the polyclonal population (**b**) adjusted to 25% brightness. **h**, Spike level *k%* of clone Sp1 in un-transduced polyclonal population (experimental scheme **a** i) compared to the value obtained from FlowJo analysis. Sp1 cells were identified by gating out the autofluorescent subpopulation. **i**, Spike level *k%* of Sp1 in 3FP+ polyclonal population (experimental scheme **a** ii) compared to the value obtained from FlowJo analysis. Sp1 cells were identified by positive DRAQ5 nuclear labeling. **j**, Spherical scatter plot of clone Sp2 (~1.8E4 cells) overlaid on the spherical scatter plot of the polyclonal population (**b**) adjusted to 25% brightness. **k**, Spike level *k%* of clone Sp2 in un-transduced polyclonal population compared to the value obtained from FlowJo analysis. **l**, Spike level *k%* of Sp2 in 3FP+ polyclonal population compared to the value obtained from FlowJo analysis. **m**, Spherical scatter plot of clone Sp3 (~1.5E4 cells) overlaid on the spherical scatter plot of the polyclonal population (**b**) adjusted to 25% brightness. **n**, Spike level *k%* of clone Sp3 in un-transduced polyclonal population compared to the value obtained from FlowJo analysis. **o**, Spike level *k%* of Sp3 in 3FP+ polyclonal population compared to the value obtained from FlowJo analysis. **p**, Spherical scatter plot of clone Sp4 (~1.3E4 cells) overlaid on the spherical scatter plot of the polyclonal population (**b**) adjusted to 25% brightness. **q**, Spike level *k%* of clone Sp4 in un-transduced polyclonal population compared to the value obtained from FlowJo analysis. **r**, Spike level *k%* of Sp4 in 3FP+ polyclonal population compared to the value obtained from FlowJo analysis. **s**, Spherical scatter plot of Clone Sp5 (~1.6E4 cells) overlaid on the spherical scatter plot of the polyclonal population (**b**) adjusted to 25% brightness. **t**, Spike level *k%* of clone Sp5 in un-transduced polyclonal population compared to the value obtained from FlowJo analysis. **u**, Spike level *k%* of Sp5 in 3FP+ polyclonal population compared to the value obtained from FlowJo analysis.

**SUPPLEMENTARY REFERENCES**

1. Arganda-Carreras, I. et al. in *Computer Vision Approaches to Medical Image Analysis 4241* (eds Beichel, R. & Sonka, M.) 85-95 (Springer Berlin Heidelberg, 2006).
